# Redox Buffering and Corrosion in Molten Chloride Salts Dictated by Chlorobasicity

**DOI:** 10.1021/jacs.6c11331

**Published:** 2026-10-16

**Authors:** Kailee Buttice, Bonita Goh, Matthew S. Emerson, Ellie Kim, Yuxiang Peng, Bobby Layne, Isabelle Baggenstoss, Shea McCarthy, George Vukovic, Changkyu Kim, Jagadeesh Sure, Sheng Dai, Céline Cannes, Sylvie Delpech, James F. Wishart, Santanu Roy, Yu-chen Karen Chen-Wiegart, Adrien Couet

**Affiliations:** † Department of Nuclear Engineering and Engineering Physics, University of Wisconsin−Madison, Madison, Wisconsin 53706, United States; ‡ Chemistry Division, 8099 Brookhaven National Laboratory, Upton, New York 11973, United States; § Department of Chemistry, University of Tennessee−Knoxville, Knoxville, Tennessee 37996, United States; ∥ Department of Material Science and Chemical Engineering, 12301 Stony Brook University, Stony Brook, New York 11794, United States; ⊥ Chemical Sciences Division, 6146 Oak Ridge National Laboratory, Oak Ridge, Tennessee 37830, United States; # 6IJCLab, CNRS/IN2P3, Université Paris-Saclay, 91405 Orsay, France; ∇ National Synchrotron Light Source-II, Brookhaven National Laboratory, Upton, New York 11973, United States

## Abstract

This study establishes an in situ synchrotron approach to directly link molten-salt chemistry, redox-buffer speciation, and alloy corrosion in chloride melts. Using a first-of-its-kind corrosion cell coupled to simultaneous X-ray absorption spectroscopy (XAS) and X-ray fluorescence (XRF), we monitor europium redox-buffer speciation and chromium dissolution during corrosion of Ni-20Cr in molten LiCl-KCl and KCl-MgCl_2_ salts. To build a mechanistic framework, we combine UV–vis optical basicity measurements, in situ electrochemistry, and molecular dynamics simulations to quantify how salt chlorobasicity reshapes europium solvation and shifts the Eu­(III)/Eu­(II) apparent potential. Increasing MgCl_2_ content drives the apparent potential of the Eu­(III)/Eu­(II) couple +0.49 V relative to LiCl-KCl and stabilizes Eu­(II)-compatible solvation environments. In situ, time-resolved Eu L_3_-edge XANES reveals progressive Eu­(III) reduction in KCl-MgCl_2_ melts, while Eu­(III) remains predominantly stable in LiCl-KCl. This behavior is consistent with a chlorobasicity-activated reaction with boron nitride (BN) cell components. In less chlorobasic melts, salt redox chemistry enables BN chlorination, allowing BN to act as a sacrificial reductant and chlorinating agent that promotes Eu­(III) to Eu­(II) conversion, whereas this pathway is not thermodynamically favored in LiCl-KCl. During active corrosion of Ni-20Cr, coupled Eu XANES and Cr XRF measurements show concurrent Eu­(III) to Eu­(II) conversion and Cr dissolution, confirming that Eu­(III) is the oxidizing species driving chromium oxidation. Together, these results demonstrate that chlorobasicity is a central control variable for redox-buffer speciation and corrosion thermodynamics, and more broadly that chlorobasicity can enable or suppress reactive pathways with structural materials in high-temperature molten salts.

## Graphical abstract

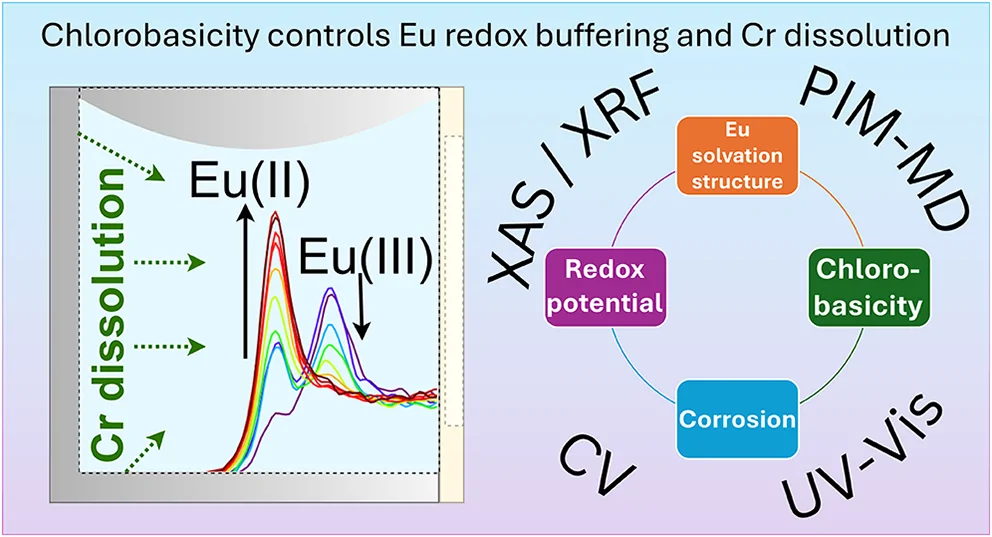

## 1 Introduction

Molten salt technologies are increasingly viewed as enabling platforms for a broad range of energy applications, from concentrated solar power to next-generation fission and fusion nuclear reactors. In particular, molten salt reactors (MSRs) offer unique advantages including high thermal efficiency, increased intrinsic safety features, and flexible fuel cycles, which have spurred rapid industrial and governmental interest.^1–10^ Molten-salt technologies are being actively pursued across both reactor and nonreactor energy applications. In nuclear energy, U.S. developers are advancing multiple molten-salt concepts, including fluoride-salt-cooled systems such as Kairos Power’s Hermes and chloride-based fast-spectrum systems, which are intended to inform future commercial reactor deployment. Beyond reactor cores, molten salts are also central to high-temperature thermal energy storage and industrial heat applications, including TerraPower’s Natrium integrated energy system. Despite this momentum, many fundamental scientific questions remain about the chemistry and material compatibility of molten salts under reactor-relevant conditions.

Among these questions, the concept of chlorobasicity, a measure of the effective Lewis basicity of chloride-based molten salts, remains relatively poorly understood. Chlorobasicity refers to the relative tendency of a chloride melt to donate through chloride ligands, and directly reflects the effective chemical potential, or activity, of chloride in a molten salt environment.^11^ It influences the speciation of dissolved metal ions, such as corrosion products, redox-active additives, which are often employed to buffer redox conditions and mitigate corrosion, and fission products, with lanthanide redox potentials known to vary systematically with the composition of the chloride solvent.^12–15^ In this way, the chlorobasicity of a molten chloride salt is analogous to the pH of an aqueous solution or the a_O2–_ in oxide melts, where pCl^–^ = −log­(a_Cl–_). However, experimental evidence on how chlorobasicity modulates soluble additive speciation, and how this speciation, in turn, affects standard reduction potentials, and possibly corrosion kinetics, is scarce. The lack of such data hinders the rational design of salt chemistries, redox buffers, and alloy compositions for improved durability in chloride molten salt environments.

Investigating these phenomena is particularly challenging because high-temperature molten salts are difficult to characterize in situ without perturbing their native speciation, are highly sensitive to impurities such as moisture and oxygen, and can cause substantial corrosion of materials under typical operating conditions. Analysis on previously melted, solid salt at room temperature faces cooling-induced structural reorganization and fails to capture the dynamic equilibrium states at operational temperatures.^16–20^ Therefore, in situ experiments are essential for elucidating the speciation of redox buffers and corrosion products in molten salts of varying chlorobasicity. Among the available techniques, synchrotron-based X-ray absorption spectroscopy (XAS), including both X-ray absorption near-edge structure (XANES) and extended X-ray absorption fine structure (EXAFS), is the most powerful tool for resolving oxidation states and coordination environments of specific elements in complex, disordered media. Recent advances have enabled in situ XAS of molten salts and dilute species,^21^ but until now, no study has demonstrated real-time, in situ tracking of both alloy corrosion and additive speciation in molten salts using synchrotron X-ray based techniques (XAS and XRF) concurrently. This represents a key capability needed to correlate speciation with corrosion dynamics and investigate how salt chlorobasicity impacts corrosion rates.

In this study, we address this gap by performing the first successful in situ corrosion experiment of bulk Ni-20Cr in chloride molten salts with additive species under in situ XAS and XRF observations. Using LiCl-KCl and KCl-MgCl_2_ with varying divalent salt concentrations, we demonstrate how salt chemistry alters Ni-20Cr corrosion behavior and governs the speciation and solvation structure of the Eu­(III)/Eu­(II) redox buffer, which is a well-characterized redox couple with relevance to fission product chemistry in MSRs.^22–24^ We first quantify how chlorobasicity varies across the salts of interest using the UV–vis response of a BiCl_3_ probe. We then determine how the Eu­(III)/Eu­(II) redox couple shifts with chlorobasicity by in situ cyclic voltammetry (CV), and we use molecular dynamics (MD) simulations to interrogate how chlorobasicity reorganizes Eu speciation and solvation structure. Building on this mechanistic picture, we perform in situ Eu L_3_-edge XANES to track Eu oxidation-state changes in salts containing a chlorobasicity-activated reductant (BN). Notably, we find that BN, which is typically treated as an inert crucible material, can act as a sacrificial reductant and chlorinating agent in melts of sufficiently low chlorobasicity, driving Eu­(III) reduction to Eu­(II). Finally, we evaluate how these coupled speciation effects influence early stage corrosion of a bulk Ni-20Cr alloy, using simultaneous Eu-XANES and Cr-XRF to link Eu redox evolution to chromium dissolution kinetics.

This work frames chlorobasicity as a controlling parameter that couples melt structure to redox thermodynamics. By reshaping speciation and solvation, the chlorobasicity of a salt shifts apparent redox potentials and dominant complexes for multivalent species (redox buffers, lanthanides, and corrosion/fission-product chlorides) so that identical nominal redox conditions can yield different chemical states and corrosion responses. The approach presented here provides the experimental-computational basis to quantify how chlorobasicity simultaneously governs melt speciation, redox thermodynamics, and corrosion behavior, enabling the rational design of salt chemistries that mitigate structural material degradation in chloride MSR environments.

## 2 Methods

### 2.1 Salt Preparation

To measure Eu speciation, in situ XAS was performed on salts containing EuCl_3_ of the following compositions: LiCl-KCl eutectic (59 mol % LiCl) and KCl-*x*MgCl_2_ (*x* = 30 and 60 mol % MgCl_2_), balance KCl. KCl-MgCl_2_ salts were prepared at the University of Tennessee. MgCl_2_ was sourced from anhydrous magnesium chloride (MgCl_2_, 99% purity), obtained from Alfa Aesar, and was further purified. The purification process included sparging with dry air at 800 °C for 2 h, followed by fractional distillation at 900 °C for 24 h, and a final sparging with chlorine gas at 800 °C for 1 h. Potassium chloride (KCl, 99.999% trace metals basis) was sourced from Sigma-Aldrich and dehydrated at 500 °C for 2 h. The purified KCl and MgCl_2_ were mixed in molar ratios of 70:30 and 40:60 in a fused silica tube fitted with a size-15 Ace-Thred fitting. The mixture was heated to 800 °C under vacuum until a homogeneous melt was obtained. Salts were sealed in an Ar glovebox in airtight jars containing desiccant and shipped to the University of Wisconsin-Madison (UW-M). LiCl-KCl salts are less prone to impurities (especially moisture) than MgCl_2_ based salts, and were prepared for the corrosion experiments by mixing commercial LiCl and KCl powders (Sigma-Aldrich, ≥99% purity), containing EuCl_3_ powder (Sigma-Aldrich, 99.9% purity) to 1 or 2 wt %, and fusing at 600 °C in a glassy carbon crucible. Fusing the salt, specifically for the corrosion experiments, was done to ensure homogenization of Eu throughout the salt prior to corrosion onset. After being prepared, salts were crushed in a mortar and pestle into a coarse powder. The purified KCl-MgCl_2_ salts were not premelted with EuCl_3_ to minimize moisture absorption. Rather, EuCl_3_ powder was added to 1 g aliquot (roughly the mass of electrolyte used in the synchrotron experiment) of each composition at room temperature and homogenized by grinding to a powder with a mortar and pestle. All operations were performed in an Ar glovebox at UW-M. Samples were packed in the glovebox, vacuum sealed, and shipped to the National Synchrotron Light Source II (NSLS-II) at Brookhaven National Laboratory to be stored within a carbon-free argon glovebox, exclusively dedicated to research with molten salt, in the Electrochemical Sample Preparation Laboratory.

### 2.2 X-ray Synchrotron Measurement Cell Design

Two custom in situ XAS measurement cells were developed to investigate: (1) the speciation of Eu-based redox buffers in chloride salts of varying chlorobasicity, and (2) the corrosion behavior of Ni-20Cr alloy under molten salt exposure. Both systems were designed to operate under high-temperature and inert gas conditions at the Beamline for Materials Measurement (BMM, 6-BM) of NSLS-II.

#### 2.2.1 Speciation Cell Design

To analyze Eu speciation in salts of varying chlorobasicity, a special cell was developed. The system consists of thin-walled (500 μm) pyrolytic BN crucibles (QS Advanced Materials) arranged in a custom-machined brass heating block. The heating block contains embedded cartridge heaters on each side and slots for BN crucibles that align with the X-ray beam. A schematic for measurements conducted within the speciation cells is shown in Figure 1a. Each BN crucible contained a different molten salt composition as detailed in Section 2.1. The entire assembly was mounted within a Kapton foil sealed chamber continuously purged with high-purity N_2_ gas to maintain an inert atmosphere during measurement. The chamber included Kapton windows (50 μm thick) aligned with the incident beam and fluorescence detector at 90°, respectively. The cells were loaded with about 1 g of salt in the molten salt Ar glovebox at the NSLS-II Electrochemical Sample Preparation Laboratory before being transported on the BMM beamline. The entire assembly is pictured in Figure S1.

**Figure 1:**
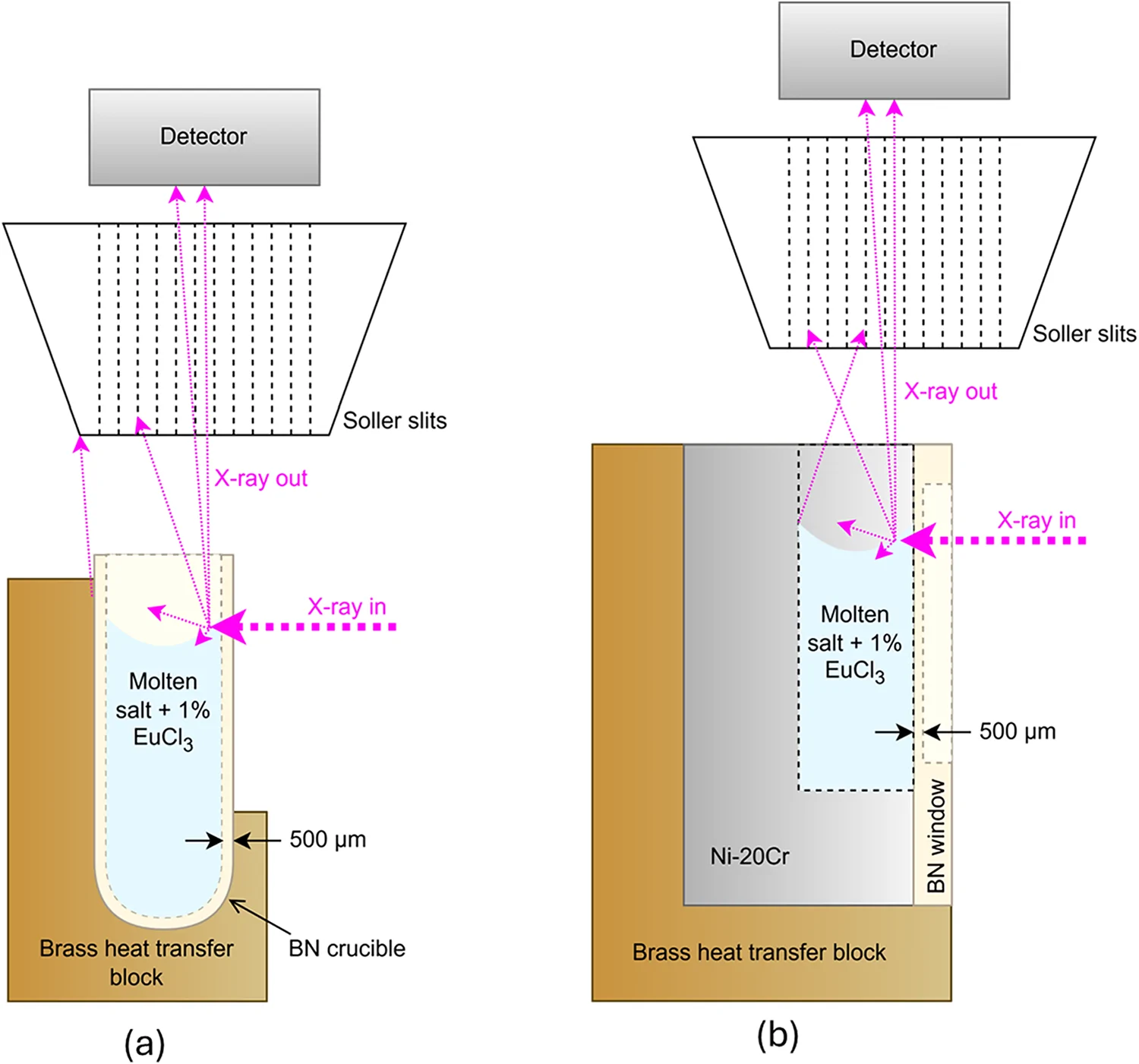
Schematic of the setup for measurements of salts contained in (a) BN crucibles for steady state Eu speciation measurements. The BN crucibles were heated simultaneously in a larger brass heat transfer block with slots for each sample. (b) Ni-20Cr blocks with BN windows for in situ corrosion measurements. Each cell loaded into the chamber was heated independently via cartridge heater in its own brass holder. Images and further details of each setup are shown in Figures S1 and S2, respectively.

#### 2.2.2 Corrosion Cell Design

A second cell type was developed to enable in situ XAS monitoring of salt chemistry during active corrosion of an alloy. These corrosion cells were constructed from Ni-20Cr alloy blocks (Goodfellow, LWH = 1″ × 0.5″ × 1″), serving as both the containment vessel and the corrosion substrate, and were milled on one face to form the shape of a three-walled rectangular body. The fourth wall, facing the X-ray beam, was made from BN (QS Advanced Materials). The pyrolytic BN window (2.5 mm thick) was bolted to the Ni-20Cr frame using brass screws. A thinned longitudinal section (15.62 mm long, 2.03 mm wide) was milled down to 500 μm at the center of the BN window to allow for X-ray beam transmission. A schematic of measurements conducted in these cells is shown in Figure 1b. Each corrosion cell was loaded with salt in the Ar glovebox at the NSLS-II Electrochemical Sample Preparation Laboratory and then mounted into a custom-designed heated holder. Thermocouples were attached directly to the Ni-20Cr substrate through drilled holes to monitor the temperature of the metal-salt interface in real-time. The holder maintained the corrosion cells at operating temperatures using the same N_2_ purged chamber as the one described for the speciation cell. The overall assembly is pictured in Figure S2.

### 2.3 Synchrotron Experimental Setup and Data Analysis

The experimental geometry was configured with a 90° angle between the incident beam and detector to capture X-ray fluorescence measurements at the BMM beamline. A double-crystal Si(111) monochromator was used. The BMM instrument uses a 3-pole wiggler source. Instrument setup includes a toroidal focusing mirror to a focused spot size of 300 μm at full width at half-maximum (FWHM). Photon flux is about 10^12^ photons per second in the delivered beam at 10 keV with a resolution of 1.3 × 10^–14^ Δ*E*/*E*. The sample was carefully positioned such that the incident X-ray beam intersected just below the molten salt surface. This ensured optimal interaction with dissolved species while minimizing salt self-absorption and scattering from the alloy substrate. Soller slits were positioned in front of the fluorescence detector to limit background scatter from secondary scattering and ensure that the detected signal originated from the molten salt region (see Figure 1).^25^ This was especially important for rejecting Cr signal from the surrounding substrate in the corrosion cell design. Prior to heating, at room temperature, only the Kα XRF emission line of potassium was visible, confirming that the beam probed the salt without signal contributions from the container alloying elements, Cr or Ni. Following heater activation, about 25 min were required for the salt temperature to reach 500 °C for LiCl-KCl and 525 °C for KCl-MgCl_2_. The slightly higher temperature for KCl-MgCl_2_ was chosen because at 500 °C the eutectic mixture containing 60% MgCl_2_ may be only partially melted. During melting, the sample must be constantly realigned with respect to the incident X-ray beam position to account for the increase in salt packing fraction as it melts. XRF and XAS measurements began immediately after thermal equilibration. In both the speciation and corrosion cell setups, XAS scans were performed for Eu (L_3_-edge, *E*_0_ = 6977 eV); additionally, in the corrosion cell setup, XRF was also performed for Cr (Kα_1_ = 5415 eV). Those were measured concurrently with each XAS scan, about every 5 min, throughout the exposure time for each alloy-salt configuration.

XAS spectra were analyzed by Athena software,^26^ while a custom MATLAB-based analysis routine (see SI Section) was developed to integrate the XRF peaks. Time-resolved fluorescence intensity was used to estimate the relative concentration of Cr dissolved in the salt over time. To account for detector drift and salt mass variation, the Cr fluorescence intensities were normalized to the height of the potassium Kα peak. Due to the time-sensitive progression of corrosion, longer acquisition time for EXAFS data was deprioritized in favor of near-edge resolution to better capture short-time scale speciation and redox evolution.

Eu XAS spectra collected during the experiments were compared against a series of reference standards for each element of interest. Spectra from EuCl_3_-doped salt samples were compared against room-temperature reference scans of Eu_2_O_3_ (representing Eu^3+^) and EuTiO_3_ (representing Eu^2+^) as well as spectra collected for EuCl_3_ and EuCl_2_ in situ in LiCl-KCl eutectic at 500 °C. These standards were used to interpret oxidation states of europium species in the salt matrices. Linear combination fitting (LCF) was performed using the Athena software package to quantify the relative contributions of reference compounds in the experimental Eu spectra.

### 2.4 UV–Vis Spectroscopy

Two compositions of purified (1-*x*)­KCl-*x*MgCl_2_ (*x* = 0.3 and 0.6) were prepared following the same procedure as detailed in Section 2.1, and ∼1 mM BiCl_3_ (Sigma-Aldrich, 99.999% purity) was added as an optical basicity probe ion to each composition. The high molar absorption coefficient of Bi^3+^, 5.4 × 10^3^ L/mol·cm in AlCl_3_–NaCl eutectic at 130 °C,^27^ at the wavelength of interest (∼330 nm) requires a very low concentration of the probe ion. A serial dilution was performed to achieve this. A KCl salt containing 400 mM BiCl_3_ was fused at 800 °C in a glassy carbon crucible and about 1 mg of the diluted KCl-BiCl_3_ was added to each 3 g sample of KCl-MgCl_2_ salt. Samples were loaded in 1 cm path length quartz cuvettes with fitted BN lids and heated to 550 °C in the UV–vis spectroscopy furnace at the UW-M. The temperature was decreased to 525 °C and allowed to equilibrate before the UV–vis spectra were collected for each sample. This was repeated at 500 °C. Measurements were collected using a Cary 4000 spectrophotometer adapted for in situ measurements via high temperature fiber optics and glovebox feedthroughs. Data processing was completed in Origin 2018. The spectrum for each salt composition without bismuth was subtracted from the Bi doped spectrum at each temperature and the isolated Bi^3+^ peak was fitted to a bi-Gaussian curve to determine peak positions. More details about the experiment can be found in Buttice et al.^28^

### 2.5 In Situ Electrochemistry

Electrochemical measurements were performed at IJCLab, Orsay (France), using an AMETEK Versastat 3F potentiostat controlled by Versastudio software with a 3-electrode setup. The working electrode was made of tungsten, the counter electrode was made of graphite, and the reference electrode was made in the laboratory and contained the AgCl/Ag redox couple (0.75 mol/kg of AgCl in LiCl-KCl). The same reference electrode was used for both salt compositions measured. The potential of the AgCl/Ag electrode was calculated to be equal to −1.033 V vs (Cl_2_/Cl^–^) at 500 °C, assuming pCl_2_ = 1 atm and a_Cl–_ = 1.^29^ The measurements were performed in an electrochemical cell containing 100 g of salt mixture prepared in a glovebox under argon by weighing and then thoroughly mixing the salts. All measurements were performed under an inert flow of Ar (Alphagaz 1 provided by Air Liquide) while the cell was in a fume hood. KCl, LiCl, MgCl_2_, EuCl_3_ were purchased from Sigma-Aldrich and their purities were respectively higher than 99.9, 99.9, 98 and 99.99%. The salt mixtures were further purified, in an Ar atmosphere, by ramping the temperature up to 300 °C for 12 h, then melting the salt and holding it under vacuum for 4 h. Next, the current proportional to the oxides was measured by running a voltammogram and AlCl_3_ was added until the current was on the order of 1 mA, which corresponds to the solubility limit of alumina. Under these conditions, no Eu­(III) precipitation (such as oxychloride) was observed, as indicated by the well-defined and reproducible voltametric response.

### 2.6 Materials Characterization

Following exposure to molten chloride salts at NSLS-II, Ni-20Cr samples were ultrasonically cleaned in deionized water for 40 min to remove any residual salt. Samples were then sectioned into cross sections and mounted in Technovit 5000 conductive epoxy for metallographic characterization. The mounted samples were ground until the metal surface was flush with the epoxy, then to 1200 grit with SiC abrasive paper and polished down to 3 μm with colloidal diamond suspension on a DiaMat wool polishing pad (Allied High Tech Products, Inc.). Scanning electron microscopy (SEM) and Energy Dispersive Spectroscopy (EDS) were conducted using a Zeiss Gemini 450 at the Nanoscale Imaging and Analysis Center at the UW-M to examine the cross sections of the salt-exposed region.

### 2.7 Molecular Dynamics Simulations and Free Energy Landscapes

Polarizable Ion Model (PIM)^30^ molecular dynamics simulations of EuCl_3_ dissolved in KCl-MgCl_2_ and LiCl-KCl molten salt mixtures were performed using *MetalWalls* simulation software.^31^ Initial configurations with ion counts in Table S1 were generated using *PACKMOL* software,^32^ ensuring random, nonoverlapping distributions of ions consistent with the target compositions. Interaction parameters for EuCl_3_ were taken from Goloviznina et al.,^33^ and combined with parameters for monovalent LiCl and KCl salts devised by Ishii et al.^34^ Interaction parameters for MgCl_2_ were taken from prior work^35,36^ and combined within the PIM formalism (see Tables S2–S3). All systems were equilibrated in the isothermal–isobaric (i.e., NPT ensemble, where *N* (number of ions), *P* (pressure), and *T* (temperature) are kept constant), following an equilibration procedure discussed previously^37,38^ for multicomponent molten chloride systems. Then, at the equilibrated averaged densities, production simulations of 1 ns duration were carried out in the canonical (*NVT*, with *V* = volume) ensemble and used to compute Eu–Cl and Eu–Mg coordination statistics and the corresponding free-energy profiles. Since no published polarizable force-field parameters are currently available for Eu^2+^ in molten chlorides, simulations involving Eu^2+^ were performed using a machine-learned interatomic potential based on the MACE framework of Batatia et al.^39–41^ using the Atomic Simulation Environment.^42^ In these simulations, initial configurations started from the final frame of the previously mentioned EuCl_3_ *NVT* production runs and further pre-equilibrated using the PIM description of EuCl_3_, after which the europium charge state was set to +2 (by removing 1 Cl^–^ ion per Eu ion and keeping the system charge neutral) and the system was then evolved for 500 ps. A corresponding frame at equilibrium density of the same equilibration procedure was then used as input for simulations using the pretrained MACE^39–41^ MP-0b3-small equivariant potential with 100 ps equilibration runs in *NVT* and production runs in *NVT* of 1 ns. For input files, see the Zenodo database submission in the Data Availability Statement. This approach provides a practical route to sample Eu^2+^ coordination environments starting from equilibrated molten-salt structures, while avoiding the current lack of published Eu^2+^ PIM parameters.^39–41,43^ The Eu^3+^ and Eu^2+^ systems were simulated separately with fixed Eu oxidation states; no electron transfer or interconversion between Eu^3+^ and Eu^2+^ occurs within these simulations. Accordingly, the simulations are used to compare the composition-dependent solvation environments of the two oxidation states, rather than to predict the Eu­(III)/Eu­(II) equilibrium ratio or redox potential.

We computed free energy profiles for two different types of ion coordination: (1) chloride and magnesium coordination around Eu^2+^ (Eu^3+^) ions and (2) chloride shared coordination between Eu^2+^ (Eu^3+^) and Mg^2+^ ions. Following our previous work,^35–38^ the chloride or magnesium coordination number around an Eu^2+^ (Eu^3+^) is defined as

1 $${\mathrm{CN}}_{\mathrm{Eu}}^{X}=\sum_{i=1}^{N_{X}}\frac{1-{\left(\frac{r_{i}}{r^{†}}\right)}^{12}}{1-{\left(\frac{r_{i}}{r^{†}}\right)}^{24}}=\sum_{i=1}^{N_{X}}f_{i}^{X}$$

Here, *N*_X_ represents either number of chlorides or number of magnesium ions. For chloride (or magnesium) coordination number, *r*_*i*_ represents the distance between the *i* ^th^ Cl^–^ (or Mg^2+^) and Eu^3+^ (Eu^2+^), while *r* ^†^ is the location of the boundary of the first chloride (or magnesium) coordination shell determined from the first minimum of the Eu^3+^(Eu^2+^)-Cl^–^ or Eu^3+^(Eu^2+^)-Mg^2+^ radial distribution functions (RDFs). In the SI, we showcase these RDFs (Figure S6), highlighting these boundaries. Note that *f*_*i*_^X^ in eq 1 ensures smooth traversals of ions across the boundary of the coordination shell, as it is a continuously deferential function. We computed the free energy profile (*W*(*CN*_Eu_^Cl^)) for the chloride coordination number from its probability distribution, *P*(*CN*_Eu_^Cl^), i.e., *W*(*CN*_Eu_^Cl^) = −*k*_B_ *T* ln­[*P*(*CN*_Eu_^Cl^)], where *k*_B_ is the Boltzmann constant. These probability distributions are accumulated from the coordination environments sampled by the Eu ions throughout the MD trajectory. Thus, the resulting free-energy profiles do not represent the free energy of the entire simulation cell or the formation free energy of a discrete Eu–Cl complex. Frequently sampled coordination environments correspond to free-energy minima, whereas less frequently sampled or short-lived coordination states occur at higher free energy. We also computed two-dimensional (2D) free energy surfaces, *W*(*CN*_Eu_^Cl^, *CN*_Eu_^Mg^) = −*k*_B_ *T* ln­[*P*(*CN*_Eu_^Cl^,*CN*_Eu_^Mg^)], from the joint probability distribution of *CN*_Eu_^Cl^ and *CN*_Eu_^Mg^, to examine how chloride and magnesium coordination numbers collectively vary around Eu^3+^ and Eu^2+^ ions.

Finally, the number of shared chlorides between a Eu^3+^(Eu^2+^) and Mg^2+^ couple is defined as

2 $$n_{\mathrm{Cl}}^{S}=\sum_{i=1}^{N_{\mathrm{Cl}}}f_{\mathrm{ij}}^{\mathrm{Cl}}f_{\mathrm{ik}}^{\mathrm{Cl}}$$

where, *f*_*ij*_^Cl^ and *f*_*ik*_^Cl^, having the same expression as given in eq 1, are respectively the contributions of the *i* ^th^ chloride to the first coordination shell of the *j* ^th^ Eu^3+^(Eu^2+^) and *k* ^th^ Mg. We computed 2D free energy surfaces as a function the Eu^3+^(Eu^2+^) and Mg^2+^ distance (*r*_Eu–Mg_) and *n*_Cl_^S^ to provide further insight into local coordination structures, namely in the form; *W­(*r_Eu–Mg_, *n*_Cl_^S^) = −*k*_B_ *T* ln­[*P*(*r*_Eu–Mg_, *n*_Cl_^S^)], where *P*(*r*_Eu–Mg_, *n*_Cl_^S^) is their joint probability.

## 3 Results

### 3.1 Europium Solvation, Speciation, and Apparent Potential in Molten Chloride Salts

#### 3.1.1 UV–Vis Spectroscopy of Molten Chloride Salts of Interest

The normalized and fitted curves for the bismuth absorption peak in each of the salt compositions (see Section 2) are shown in Figure 2a. Here, Bi^3+^ behaves as a dilute spectator ion which reports the extent of chloride availability in a melt via the wavelength of its s-p transition, which increases in response to electron donation by Cl^–^. The resulting difference in Bi^3+^ peak energies between a sample salt and a reference salt is used to calculate an optical basicity (OB) value, which quantifies the relative difference in chlorobasicity between the sample and reference salt.^44,45^ Measuring OB as a proxy for Lewis basicity is a well-established method. Originally developed by Duffy and Ingram in the 1970s,^44,46^ it has been correlated extensively to the physicochemical properties of oxide-based melts,^47–50^ and more contemporarily extended to halide salt systems.^51–54^ It reflects the electron-donating power of the melt, which is conceptually tied to chlorobasicity, i.e., the availability of Cl^–^ to provide electron density. While the two are not numerically equivalent, the trend between them (higher optical basicity ↔ higher chlorobasicity) provides an operational way to rank the relative basicity of melts of different compositions.

**Figure 2:**
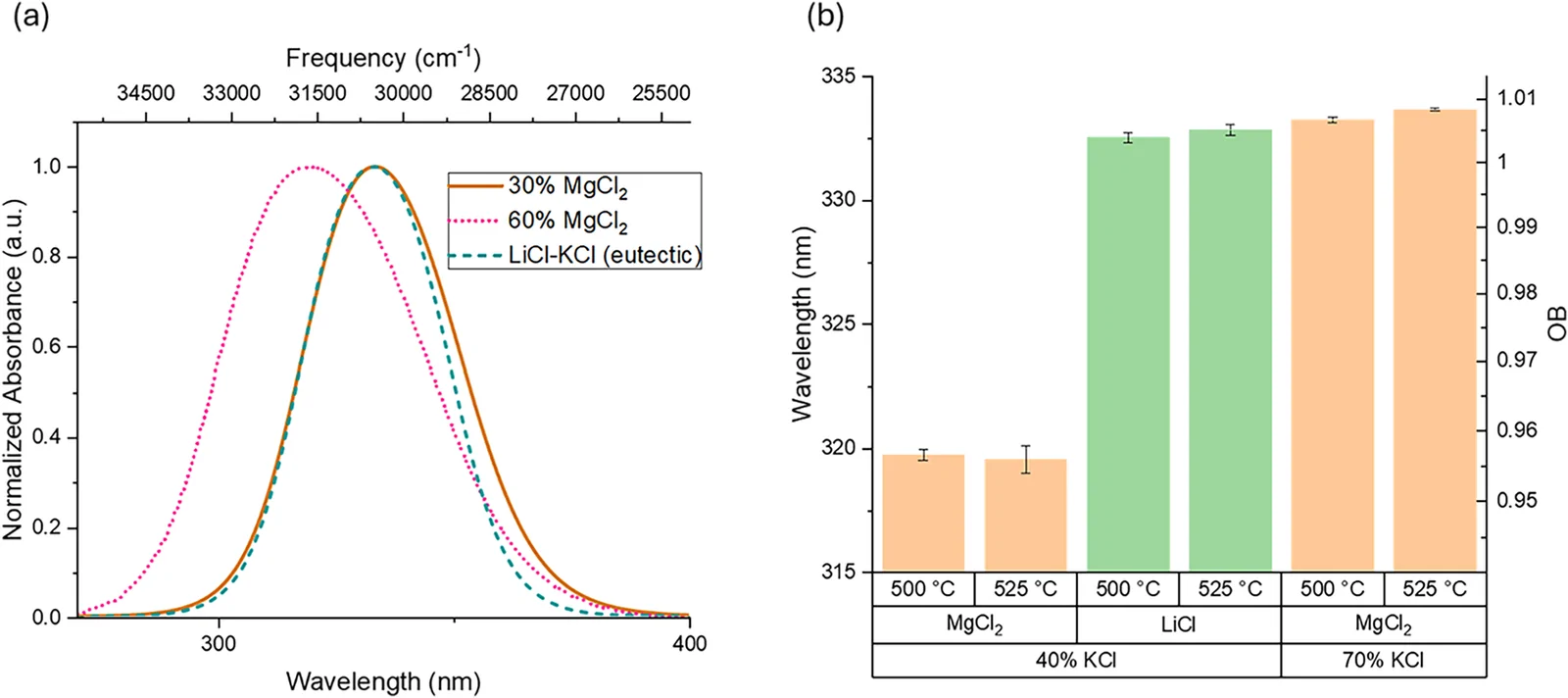
Optical basicity of chloride molten salts determined from Bi^3+^ UV–vis absorption: (a) Gaussian curves fitted to blank-subtracted experimental data at 525 °C and normalized in KCl-MgCl_2_ (30 and 60 mol %) at 525 °C. (b) Wavelength and calculated optical basicity as a function of salt composition at each temperature.

There is a clear reduction in peak wavelength as the amount of MgCl_2_ in the salt increased and the plotted calculated optical basicity and associated wavelength as a function of composition is shown in Figure 2b. The results show a decrease in the amount of energy required for the 1S_0_ → 3P_1_ transition, which indicates a decrease in salt chlorobasicity. More detailed, composition and temperature dependent optical basicity measurements across several binary chloride salts systems is reported by Buttice et al.^28^ This behavior is consistent with the expected chemical trends–metals with greater charge density and smaller size have a more significant contribution to the nephelauxetic effect–as well as with recent electrochemically measured chlorobasicity results in MgCl_2_-bearing salts by Maltsev et al.^55^ Mg^2+^, having smaller ionic radius than K^+^ and higher valency than both K^+^ and Li^+^, is more likely to polarize Cl^–^ anions, reducing the extent to which they interact with the Bi^3+^ probe ion. This corresponds to the definition of OB of a molten oxide glass given by Duffy as the average polarization state of the probe ion’s nearest neighbors.^56^ It should be noted that electrochemical measurements with a Cl_2_/Cl^–^ reference electrode would provide a more direct, quantitative measure of Cl^–^ activity, as has been done effectively by Maltsev et al.^55^ However, this represents a substantial separate experimental effort beyond the scope of the present study and is better suited for future work.

#### 3.1.2 In Situ Electrochemistry of Europium in Molten Chloride Salts of Interest

The potential measurement results are shown in Figure 3. These measurements were performed in molten salts containing 5 × 10^–2^ mol/kg of EuCl_3_. A reversible soluble/soluble system was observed, and the apparent potential *E*_app_ of the Eu­(III)/Eu­(II) system was deduced from the measured potential on the cyclic voltammetry (CV) curves (see Figures S3, S4) using the Nernst equation

3 $$E=E^{{}^{\circ}}+\frac{2.3\mathrm{RT}}{F}\log\frac{\gamma \left(\mathrm{Eu}\left(III\right)\right)\cdot x\left(\mathrm{Eu}\left(III\right)\right)}{\gamma \left(\mathrm{Eu}\left(II\right)\right)\cdot x\left(\mathrm{Eu}\left(II\right)\right)}$$

4 $$E_{app}=E^{{}^{\circ}}+\frac{2.3\mathrm{RT}}{F}\log\frac{\gamma \left(\mathrm{Eu}\left(III\right)\right)}{\gamma \left(\mathrm{Eu}\left(II\right)\right)}$$

**Figure 3:**
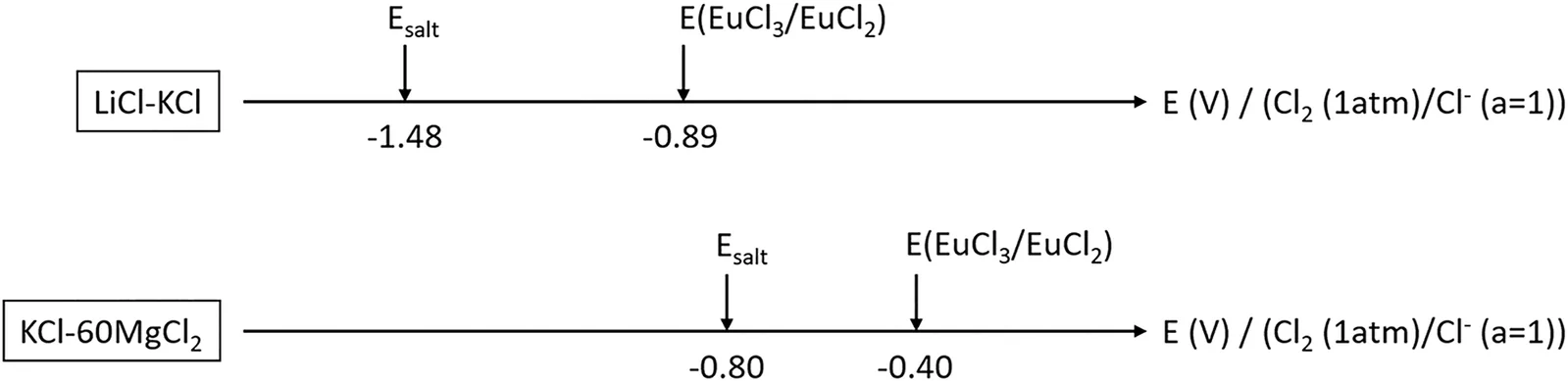
Schematic positions of the salt redox potential (*E*_salt_) and the Eu^3+^/Eu^2+^ apparent redox potential in LiCl-KCl and KCl-MgCl_2_ (60 mol % MgCl_2_) at the experimental temperature. Potentials are reported on the chlorine scale relative to (Cl_2_ (1 atm)/Cl^–^ (*a* = 1)).

In these relations, *E* is the experimental potential, *E*° is the standard potential of Eu­(III)/Eu­(II) redox system, γ­(Eu­(III)) and γ­(Eu­(II)) are the activity coefficients of Eu­(III) and Eu­(II) respectively, *x*(Eu­(III)) and *x*(Eu­(II)) the mole fraction of the respective Eu oxidation states. *R* is the ideal gas constant, *T* the temperature and *F* the Faraday constant.

The chlorobasicity is characterized by the chloride activity in a molten salt. In these systems, chloride activity should be understood as an effective thermodynamic measure of chloride availability (or donor strength), rather than as a literal concentration of free Cl^–^ ions. Here, effective chloride activity is used as a descriptor of chlorobasicity rather than as an explicit term in the Eu­(III)/Eu­(II) Nernst equation. Changes in chlorobasicity alter the solvation environments and therefore the activity coefficients of the two oxidation states, which determine the composition dependence of the apparent potential. On a chlorobasicity scale, the reference is given by alkali metal chloride salts, in which Cl^–^ anions are assumed to be only weakly polarized by the alkali metal cations so that they retain maximal donor strength toward solute cations. Accordingly, LiCl-KCl is taken here as the reference state, for which the effective chloride activity is set to 1. For salts consisting of an alkali metal and another element, such as an alkaline earth metal, the effective activity of chlorides is less than 1. Composition-dependent changes in chlorobasicity can modify the solvation environment and consequentially the activity coefficients of dissolved europium species. Because the apparent Eu­(III)/Eu­(II) potential depends on the ratio of these activity coefficients, differences in the stabilization of the two oxidation states with melt composition can produce larger shifts in the measured potential than can be accounted for by the directly measured changes in chloride activity (Maltsev et al.).^55^ Thus, the observed solvent-dependent redox potential is interpreted in terms of the ratio of Eu­(III)/Eu­(II) activity coefficients, which are modulated by the chlorobasicity of the melt. As discussed later, the MD results suggest that Eu­(III)- and Eu­(II)-bearing species do respond differently to melt composition, providing further insight into the composition- and oxidation-state dependent changes in Eu–Cl coordination and surrounding melt structure.

Across the two molten chloride salt compositions tested by electrochemistry (LiCl-KCl eutectic and KCl-MgCl_2_ (60 mol % MgCl_2_)), Figure 3 shows that solvent chlorobasicity shifts the salt redox potential measured on the W electrode, as well as the Eu­(III)/Eu­(II) apparent potential. Such solvent-dependent shifts are consistent with previous electrochemical studies showing that the formal Eu­(III)/Eu­(II) potential varies with the composition and cationic character of alkali chloride melts.^15^ Because both Eu­(III) and Eu­(II) species are present in the melt, as indicated by the reversible anodic and cathodic responses of comparable magnitude in the cyclic voltammograms (Figures S3, S4), the Eu­(III)/Eu­(II) redox potential was estimated experimentally from the CVs as the apparent equilibrium potential of the reversible soluble/soluble couple, from the midpoint of the zero-current crossover region between the anodic and cathodic branches (Figure S3), or the mean potential between the anodic and cathodic peaks (Figure S4). The two methods produce apparent potentials that agree within ±10 mV. The values reported below correspond to the latter method. Note that estimation of redox potential from a CV is not the most rigorous method available. More accurate methods may involve equilibrium potentiometry, in which the Eu­(III)/Eu­(II) ratio is controlled to verify the Nernstian slope of *E* vs ln­[Eu­(III)/Eu­(II)], so *E*_0_ may be extracted from the intercept. Alternatively, more precise analysis of the CVs may be done after correction for the Eu­(III)/Eu­(II) diffusion coefficient ratio. For the scope of this work, simpler estimation methods remain sufficient, as the relative shift in apparent potential between the two salts measured clearly indicates a notable difference in the behavior of the soluble species. This difference in behavior can be attributed to the difference in chlorobasicity between the two salts.

In LiCl-KCl, the salt redox potential measured on the W electrode lies near −1.48 V/Cl_2_ (relative to the Cl_2_/Cl^–^ couple), and the Eu^3+^/Eu^2+^ couple is estimated from the CV at about −0.89 V/Cl_2_. The separation between these two markers is 0.59 V. In KCl-MgCl_2_ (60 mol % MgCl_2_) the melt is less chlorobasic, with a salt redox potential near −0.80 V/Cl_2_ and a Eu^3+^/Eu^2+^ apparent potential near −0.40 V/Cl_2_, i.e., shifted more positive relative to LiCl-KCl. The corresponding separation is 0.40 V. Since an oxidant becomes stronger as its reduction potential shifts more positive, Eu­(III) is thermodynamically more inclined to accept an electron in the MgCl_2_-rich melt than in LiCl-KCl, while preserving the relative ordering of the salt redox and Eu­(III)/Eu­(II) potentials.

#### 3.1.3 Molecular Dynamics Simulations of Eu in Molten Chloride Salts

To elucidate how salt chlorobasicity influences Eu speciation and solvation structure at the molecular level, we employed MD simulations to characterize the composition-dependent free-energy landscapes of their local coordination environments. The two oxidation states are simulated independently and do not interconvert; therefore, the MD results are interpreted as structural comparisons between Eu­(III) and Eu­(II), rather than predictions of their equilibrium redox ratio. The simulations resolve how variations in salt composition reorganize both the Eu first-shell chloride coordination and the surrounding cationic environment, which together define the local structural response of the melt to changes in effective chloride activity. In Figure 4, the free energy profiles as a function of the Eu–Cl coordination number show that Eu samples distinct coordination states in KCl-MgCl_2_, depending on the Eu charge state and MgCl_2_ mol %. For instance, Eu^3+^ coordinates with six chlorides at a lower MgCl_2_ mol %, while doubling the MgCl_2_ mol % allows Eu^3+^ to adopt two distinct thermodynamically metastable coordination states, such that a small fraction of the six-coordinate state can now transit to the high-energy seven-coordinate state by overcoming a barrier larger than the thermal energy. The solvent composition effect is more dramatic for Eu^2+^. It prefers only the six-coordinate state exclusively at the lower MgCl_2_ mol % concentration, while it adopts exclusively the seven-coordinate states at the higher MgCl_2_ mol % concentration. These findings are consistent with our previous studies^35–38^ of molten salts comprising multivalent cations, where their coordination numbers were found to increase to higher values with higher multivalent cation concentrations.

**Figure 4:**
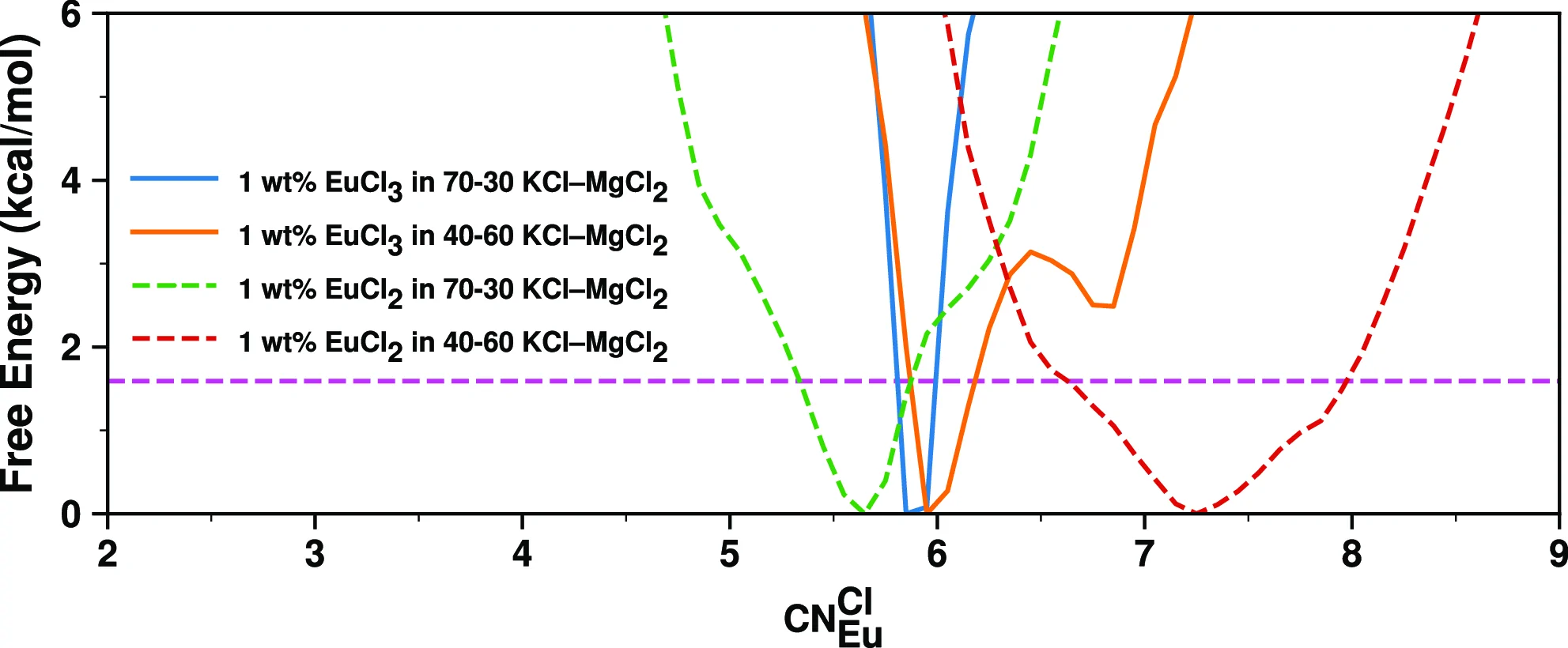
Free energy as a function of the Cl^–^ coordination number around europium ions. Solid lines correspond to Eu^3+^ and dash–dot lines to Eu^2+^; colors denote different salt compositions. The thermal energy scale (*k*_B_ *T* = 1.59 kcal mol^–1^) is indicated by the horizontal magenta line.

The solvent composition and charge state-dependency of Eu–Cl coordination is suggestive of strongly coupled collective rearrangements of the melt structure around Eu that goes beyond the first coordination shell. This extended, collective ion coordination metric can serve as a structural proxy for how chlorobasicity manifests locally in the melt. We anticipate that changes in chlorobasicity due to composition changes can alter Eu behavior by primarily reshaping the coupled first (anionic) and second (cationic)-sphere solvation landscape, rather than by modifying the first coordination shell in isolation. To investigate this hypothesis, we first consider the fact that Mg cations form networks and Eu cations join the network by sharing their chlorides with Mg. We quantify Eu’s compatibility with Mg networks using the 2D free energy surfaces (see Figure 5) describing the number of chlorides simultaneously coordinated to Eu and Mg, namely *n*_Cl_^s^. Nonzero *n*_Cl_^s^ at short Eu–Mg separation indicates that Eu is connected to Mg through bridging chlorides, i.e., partially integrated into the Mg–Cl connectivity. In MgCl_2_-rich environments, this shared-anion metric provides a direct structural proxy for chlorobasicity. Specifically, less chlorobasic melts favor chloride participation in Mg-centered motifs, and we find that Eu^2+^ exhibits greater propensity to occupy these shared-chloride environments than Eu^3+^. This observation is indicative of enhanced compatibility of Eu^2+^ with Mg networks, which is arising from the enhanced ability of Eu^2+^ to accommodate more chlorides within its first shell in Mg-rich environments.

**Figure 5:**
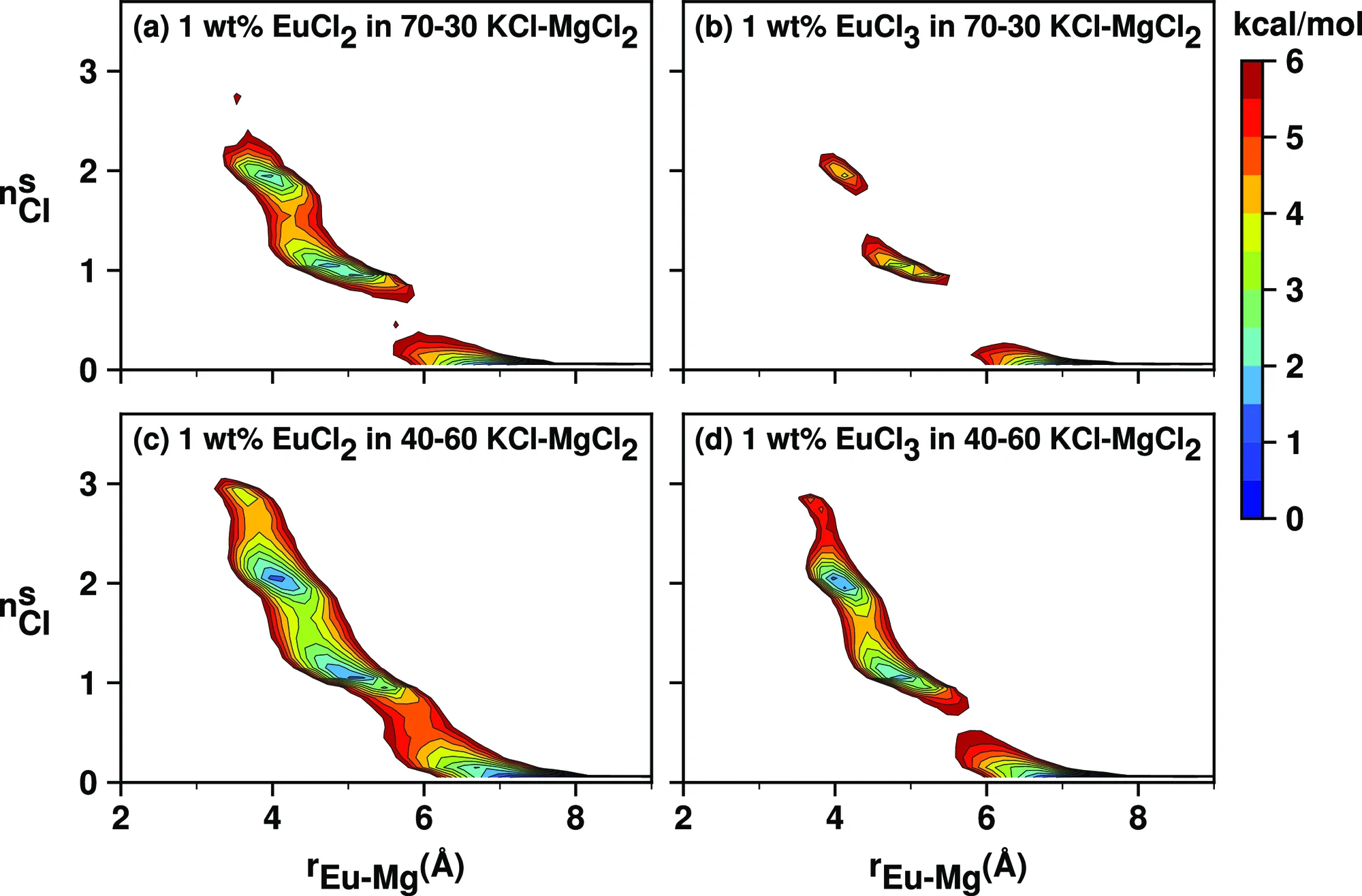
2D free energy surfaces correlating the number of shared chlorides with the distance between Eu and Mg ions for EuCl_2_ (left) and EuCl_3_ (right) in KCl-MgCl_2_ mixtures with compositions of (a, b) 70-30 and (c, d) 40-60, corresponding to molar percent.

Supporting the discussion above, the coupling between the chloride coordination and the surrounding cationic environment is made explicit in the 2D free energy surfaces shown in Figure 6. These surfaces reveal that coordination states with similar Eu–Cl CNs can differ substantially in free energy depending on the local Mg population. In MgCl_2_-rich melts, Eu^2+^ samples regions of the free energy surface associated with higher Mg coordination more readily than Eu^3+^, indicating that the reduced ion is more compatible with Mg-rich local environments. Conversely, Eu^3+^ exhibits a more restricted free energy basin that disfavors configurations with high local Mg density, consistent with a stronger sensitivity to the surrounding cationic field.

**Figure 6:**
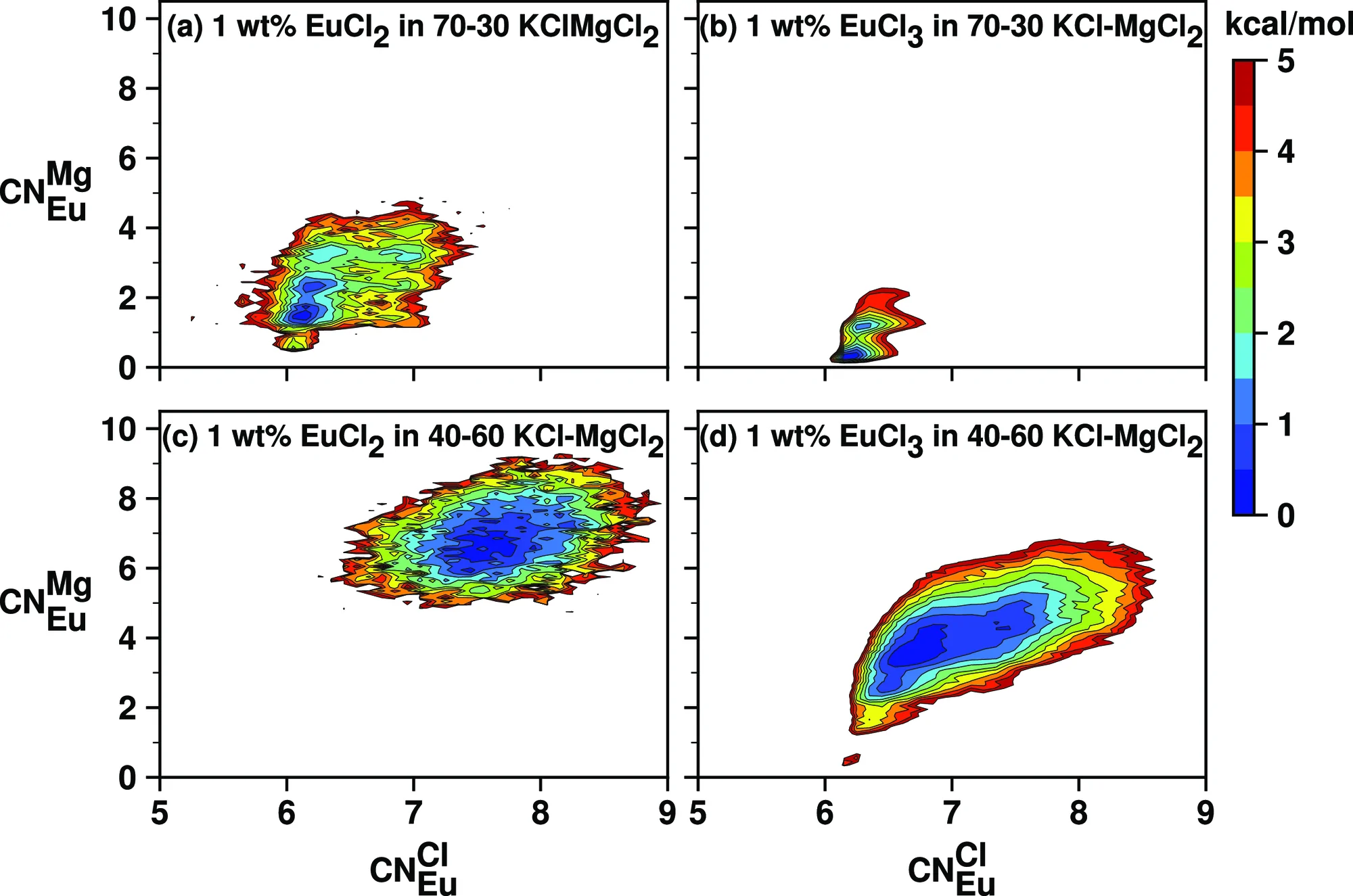
2D free energy surfaces correlating the number of Mg ions around Eu ions with the number of chloride ions around Eu ions for EuCl_2_ (left) and EuCl_3_ (right) in KCl-MgCl_2_ mixtures with compositions of (a, b) 70–30 and (c, d) 40–60, corresponding to molar percent.

### 3.2 In Situ Corrosion Evolution and Postexposure Analysis of Ni-20Cr in Molten Chloride Salts

#### 3.2.1 Europium XANES Standards

The lanthanide (Ln) L_3_-edge XANES technique is a powerful tool for probing Ln oxidation states, as it can clearly distinguish between Ln^3+^ and Ln^2+^ species based on a characteristic shift in the absorption edge of approximately 7–8 eV.^57–59^ The L_3_-edge XANES data of the four Eu standards used in this study is shown in Figure 7. Normalized Eu L_3_-edge XANES spectra of EuCl_2_ and EuCl_3_ dissolved in molten LiCl-KCl at 500 °C were compared to reference spectra of well-characterized Eu_2_O_3_ (Eu^3+^) and EuTiO_3_ (Eu^2+^) powders. EuCl_2_ dissolved in LiCl-KCl exhibits an intense lower-energy white-line feature centered around 6974 eV, closely matching the EuTiO_3_ standard, while EuCl_3_ shows a dominant higher-energy white-line feature near 6984 eV, consistent with the Eu_2_O_3_ standard reference. These white-line positions and intensities, notably the clear separation between the Eu^2+^ and Eu^3+^ edge features, highlight the ability of XANES to resolve the oxidation state of Eu in molten salt environments. The spectral shapes are also consistent with previous observations that Eu^2+^ species exhibit sharper, more intense white lines, due to simpler final-state interactions and lower core–electron binding energies, while Eu^3+^ spectra show broader, higher-energy white-line features because of stronger 4f-5d coupling and greater effective nuclear charge.^60^

**Figure 7:**
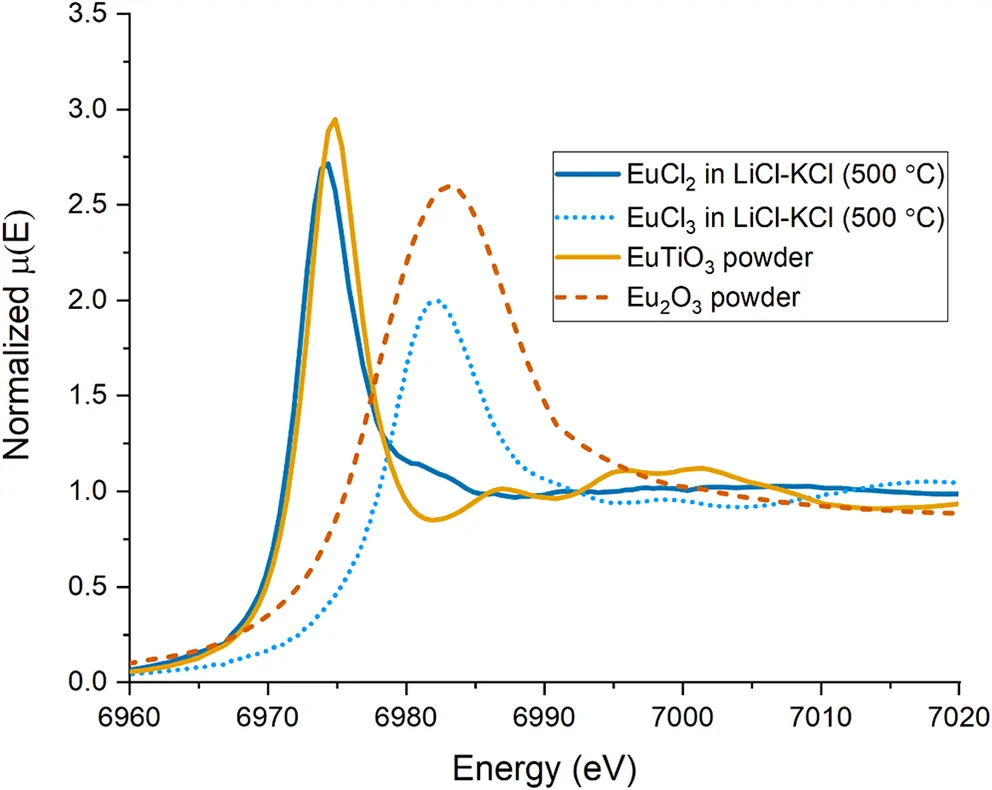
Normalized XANES spectra at the Eu L_3_-edge for EuCl_3_ and EuCl_2_ in LiCl-KCl at 500 °C, compared with reference powders of Eu_2_O_3_ and EuTiO_3_.

#### 3.2.2 Steady State Eu Speciation in Molten Chloride Salts of Interest

Figure 8 presents the time-resolved Eu L_3_-edge XANES spectra for EuCl_3_ dissolved in KCl-30%MgCl_2_, and corresponding linear combination fitting (LCF) results for EuCl_3_ dissolved in the three different molten chloride salts (see Section 2). Spectra were collected in situ using the speciation cell design and monitored over time until a steady-state speciation was reached, typically within 30–40 min. The normalized XANES spectra were then quantitatively analyzed using the ATHENA software package,^26^ employing EuCl_2_ and EuCl_3_ (in LiCl-KCl eutectic) standards, detailed in Section 3.2.1, for LCF. The results clearly show that the final europium oxidation state depends strongly on molten salt chemistry. In LiCl-KCl, the spectrum remains dominated by Eu^3+^ features, indicating limited change in Eu chemistry. In contrast, Eu^2+^ becomes increasingly stabilized as the MgCl_2_ content in the salt increases, with the KCl-60 mol % MgCl_2_ salt exhibiting predominantly Eu^2+^ features in the final spectrum. The underlying reasons for the change in Eu oxidation state in the different salts will be developed in more detail in the Discussion section.

**Figure 8:**
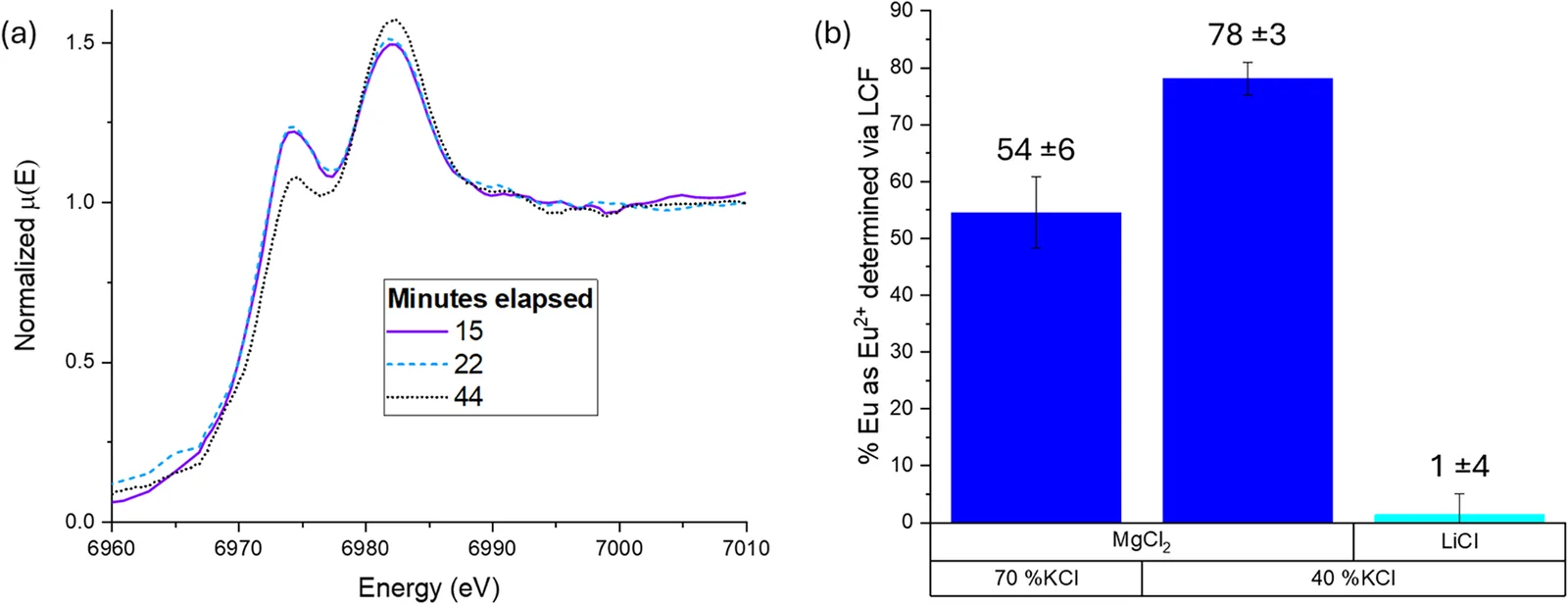
Time-resolved Eu XANES analysis in speciation cell. (a) Normalized Eu L_3_-edge XANES spectra of 1 wt % EuCl_3_ in KCl-30%MgCl_2_ after 15, 22, and 44 min (b) Quantification of %Eu as Eu^2+^ in the salt for each salt of interest determined using linear combination fitting.

#### 3.2.3 In Situ Ni-20Cr Alloy Corrosion: Cr X-ray Fluorescence and Eu XANES

Figure 9 presents the time-resolved Cr Kα XRF intensity (Figure 9a) and Eu L_3_-edge XANES spectra (Figure 9b) collected during in situ corrosion of Ni-20Cr in molten KCl-30%MgCl_2_ salt containing 1 wt % EuCl_3_ at 525 °C. As corrosion progresses, the Cr fluorescence signal steadily increases, indicating dissolution of Cr into the salt. Simultaneously, the Eu L_3_-edge spectra show a shift from Eu^3+^ to Eu^2+^ features over time, consistent with the progressive reduction of Eu­(III). These concurrent observations suggest a redox reaction between the dissolving alloy and the redox buffer, where metallic Cr is oxidized to Cr^2+^ while Eu^3+^ is reduced to Eu^2+^, as described by the reaction in eq 5

5 $$2{\mathrm{Eu}}^{3+}+\mathrm{Cr}\to 2{\mathrm{Eu}}^{2+}+{\mathrm{Cr}}^{2+}$$

**Figure 9:**
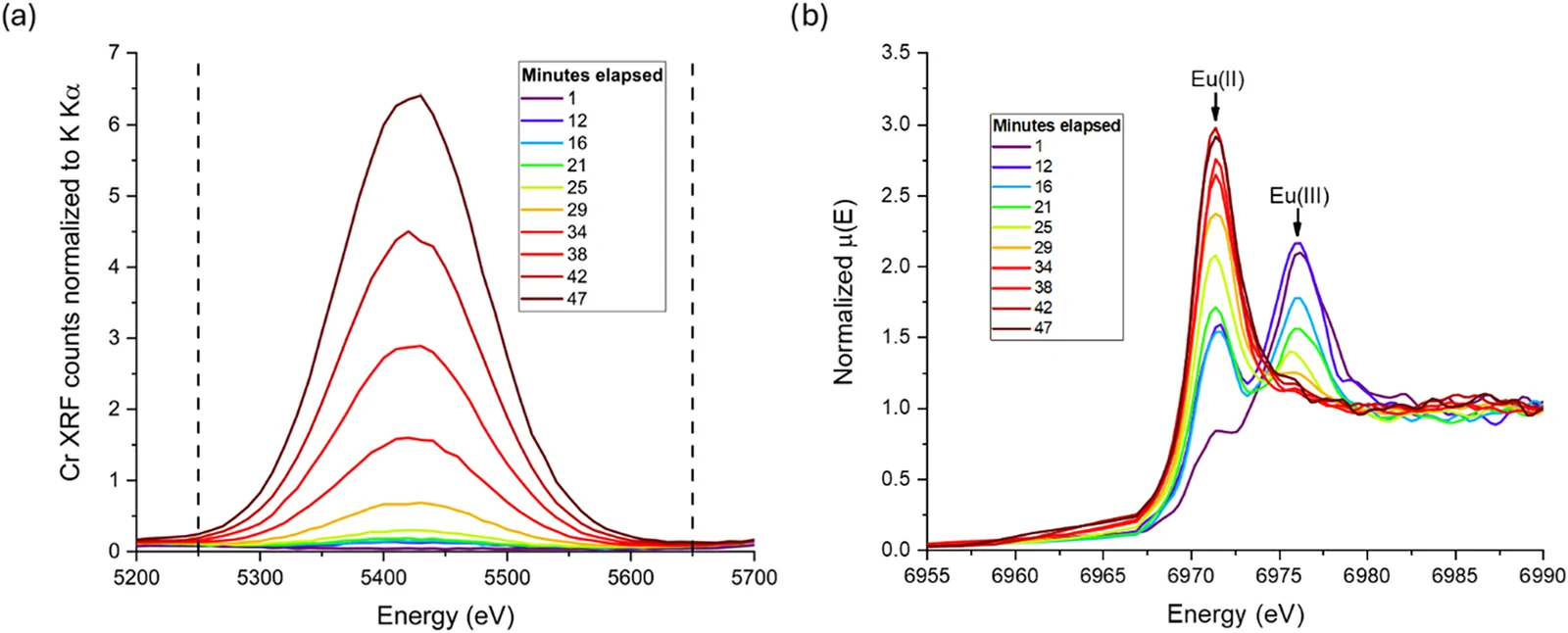
Coupled evolution of chromium dissolution, and europium redox speciation during in situ corrosion of Ni-20Cr in KCl-30%MgCl_2_ molten salt containing 1 wt % EuCl_3_ at 525 °C. (a) Time-resolved in situ XRF signal of Cr Kα1 counts, normalized to K Kα. Dotted lines represent integration bounds for Cr counts in Figure 10. (b) Corresponding Eu L_3_-edge XANES spectra collected at each time point, indicating a progressive shift in edge position and white-line intensity consistent with Eu oxidation state change.

The data highlights the interplay between corrosion and europium speciation, providing real-time evidence of the coupling between alloy degradation and the redox chemistry of the molten salt environment. Notably, qualitative comparison of the Eu XANES data with the earlier speciation experiments (Figure 8) shows more pronounced Eu^2+^ features over time in the presence of Ni-20Cr corrosion. This suggests that corrosion is driven by oxidation of the alloy by Eu­(III), which is simultaneously reduced to Eu­(II), leading to an excess Eu­(II) signal compared with equilibration with the salt alone in the speciation cell.

Figure 10 presents the quantitative results from LCF of Eu L_3_-edge XANES data alongside the Cr Kα XRF signal, tracking corrosion and Eu oxidation state evolution during exposure of Ni-20Cr to chloride molten salts. In KCl-MgCl_2_ (Figure 10a) and LiCl-KCl eutectic salts (Figure 10b), the Eu oxidation state shifts from Eu^3+^ to nearly 100% Eu^2+^ within 60 to 120 min. This transformation aligns closely with the progressive detection of Cr in the salt, suggesting again that Eu^3+^ acts as an oxidant and is reduced to Eu^2+^ via the corrosion of Cr, following eq 5. When comparing the effect of the salt composition, in MgCl_2_-containing salts, the rate of Cr corrosion is faster for the 30 mol % MgCl_2_ salt compared to the 60 mol % MgCl_2_ salt, as reflected by the higher Cr XRF intensity observed during the experiment. For the LiCl-KCl salt, a control test without EuCl_3_ shows negligible Cr XRF signal, supporting the role of Eu as the primary corrosion mediator and confirming limited interference from other possible redox-active species such as oxygen and moisture impurities. The mismatch between the shapes of the Cr counts and %Eu­(II) curves in Figure 10(a,b) appears to contradict this point, but can be attributed to several factors. Cr is only released once sufficient corrosive attack has occurred, producing a lag before dissolved Cr becomes detectable, whereas Eu­(III)/Eu­(II) equilibration responds more rapidly to the local environment. The Eu­(III)/Eu­(II) fraction is obtained from linear combination fitting of edge-normalized XANES spectra and is therefore insensitive to fluctuations in absolute count rate, whereas the Cr Kα signal is an absolute fluorescence intensity and is correspondingly more sensitive to such fluctuations. Chromium Kα is also inherently harder to quantify than Eu by XRF, owing to its stronger self-absorption of the relatively soft Cr Kα photons in the salt and the correspondingly lower signal-to-noise, especially at the low Cr concentrations expected relative to the 1 wt % Eu loading. Finally, the beam probes only a thin salt volume near the surface (see Figure 1) to limit self-absorption, and the Soller slits further limit the detected photon efficiency to limit substrate interference. Chromium released at the corrosion interface must diffuse into this detection region unlike Eu, which was homogeneously distributed in the salt prior to melting.

**Figure 10:**
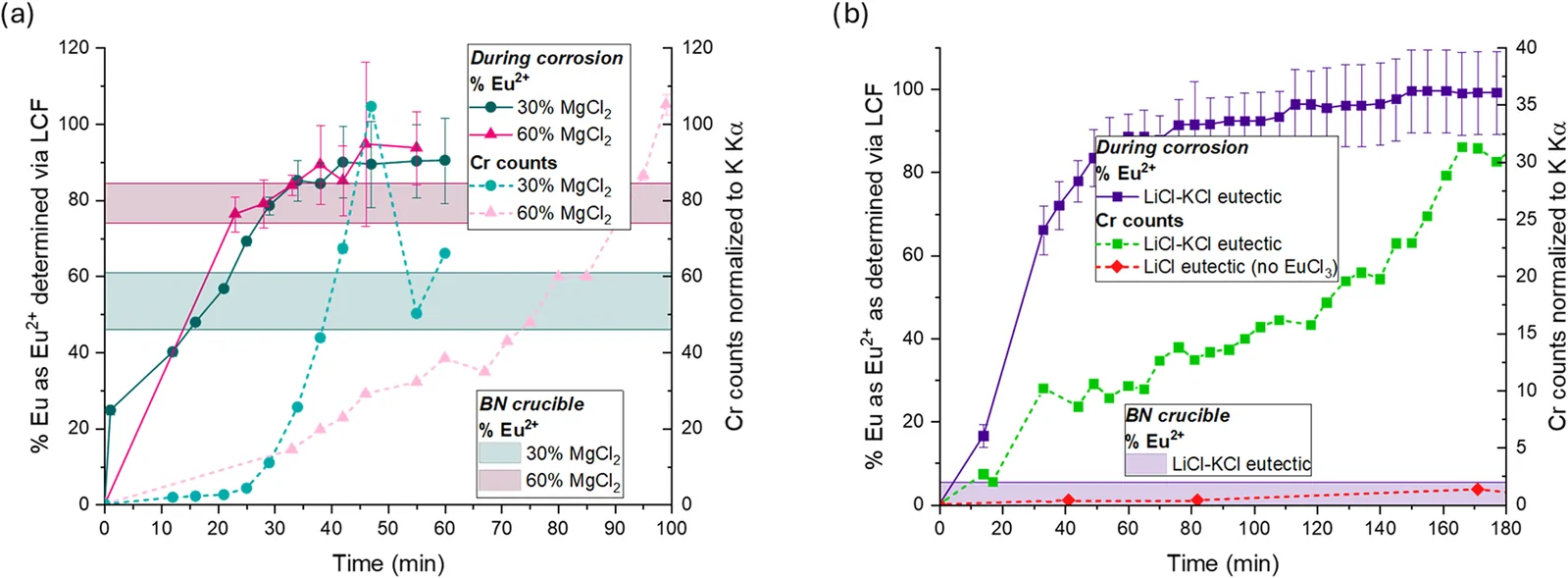
Time evolution of %Eu as Eu^2+^ and Cr Kα XRF signal normalized to K Kα during Ni-20Cr alloy exposure to molten salts: (a) KCl–*x*MgCl_2_ salts containing 1 wt % EuCl_3_ varying MgCl_2_ concentrations at 525 °C and (b) LiCl-KCl salts containing 1 wt % EuCl_3_ at 500 °C. Panel (b) includes Cr XRF data for an undoped salt with no europium as well. The data for Eu speciation in the BN crucible is reproduced from Figure 8 for easy comparison.

An additional contribution specific to the MgCl2-rich melts is that BN itself can reduce Eu­(III) under low chlorobasicity conditions, as demonstrated in our speciation cell experiments; a portion of the early Eu­(III) → Eu­(II) conversion in the corrosion cell therefore need not be stoichiometrically matched by Cr release, which further relaxes the expectation that the two curves should coincide in shape.

Interestingly, a transient decline in Cr XRF signal is observed in the 60 mol % MgCl_2_ salt. This decline may reflect the onset of chromium oxide precipitation (Cr_2_O_3_ or MgCr_2_O_4_), enabled by residual oxide impurities that are more abundant in MgCl_2_-rich melts. Precipitation and settling of Cr-bearing solids would temporarily deplete dissolved Cr from the near-surface volume probed by the beam, reducing the Cr Kα signal until continued corrosion resupplied dissolved Cr to the melt. This effect would be compounded by the low Cr concentration and reduced signal-to-noise at the Cr Kα edge, making these measurements particularly sensitive to such fluctuations.

#### 3.2.4 Postcorrosion Characterization of Ni-20Cr in LiCl-KCl Salts

Figure 11 presents cross-sectional SEM images and associated EDS line profiles of Ni-20Cr samples exposed to LiCl-KCl salts with increasing concentrations of EuCl_3_ (0, 0.6, and 2 wt %), using the same corrosion XAS cell geometry (Figure 1b), highlighting the evolution of elemental composition near the alloy-salt interface after corrosion. Note that the concentrations of EuCl_3_ used in the corrosion experiments described in the rest of this paper (0, 1 wt %) vary from the concentrations used for the samples characterized by SEM. These tests were conducted at two separate beamtimes. Following the corrosion experiments with 2 wt % EuCl_3_, it was determined that corrosion proceeded too quickly to acquire sufficient time-dependent data on Eu speciation and Cr dissolution, so the remainder of experiments were conducted with an intermediate 1 wt % EuCl_3_ concentration. While no visible corrosion attack is observed on the SEM alloy cross-section, likely because of the short exposure time of only a few hours, the EDS profiles close to the metal surface (up to 1 μm) reveal that as the EuCl_3_ concentration increases, the depth of Cr depletion into the alloy visibly grows, indicating more extensive corrosion. The increased depth of Cr loss from the alloy further supports the interpretation that Eu^3+^ acts as an oxidizing agent, mediating the dissolution of Cr within the alloy microstructure.

**Figure 11:**
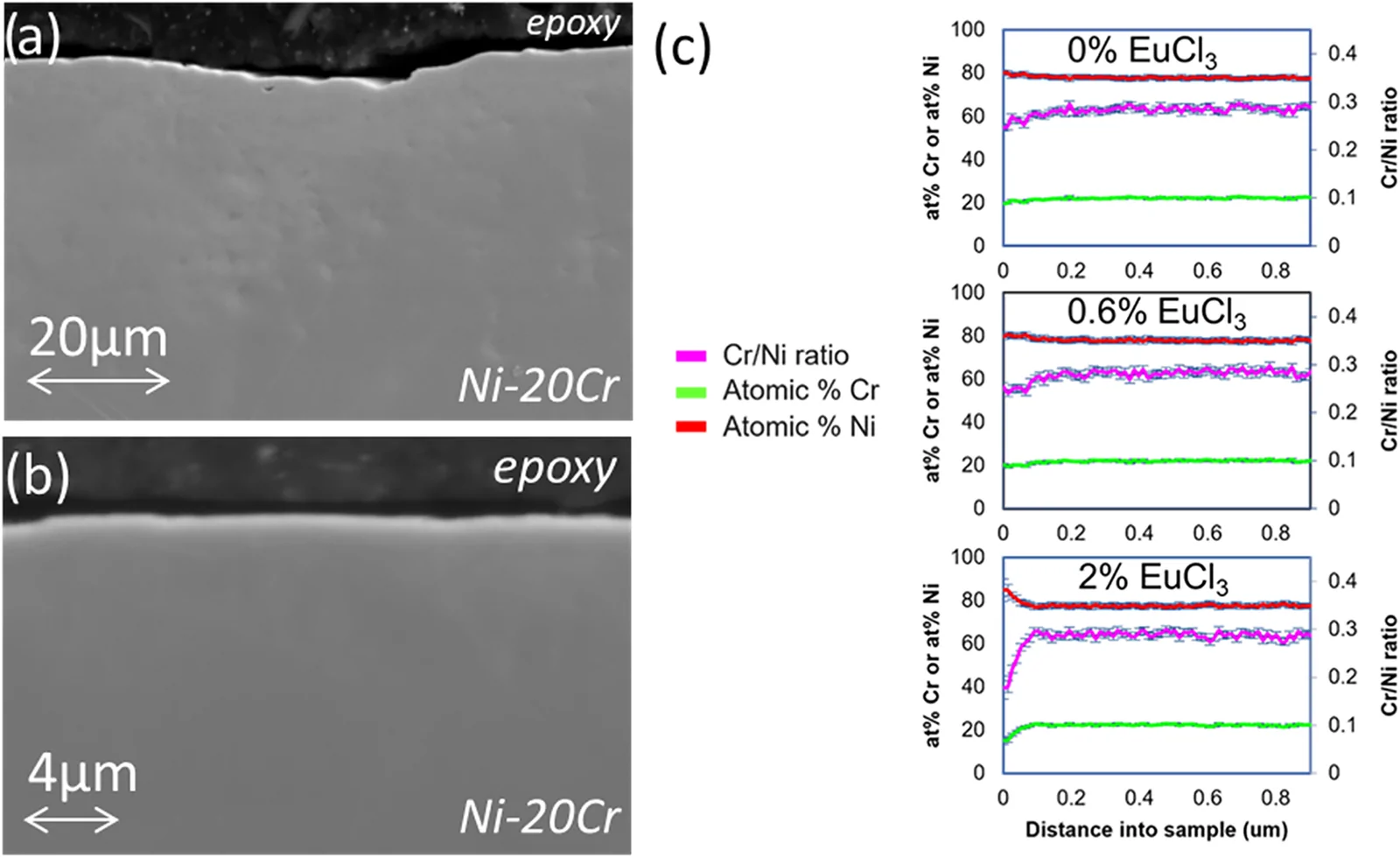
SEM cross-sectional images of Ni-20Cr after exposure to molten LiCl–KCl salt containing 2%EuCl_3_ at 500 °C (a) 3000X, (b) 10,000X, (c) Depth profiles of atomic percent Cr and Ni, and Cr/Ni ratio, measured by EDS in Ni-20Cr alloy after exposure to molten LiCl–KCl salt containing varying concentrations of EuCl_3_ (0%, 0.6%, and 2%) at 500 °C.

## 4 Discussion

### 4.1 Chlorobasicity Effect on Lanthanide Solvation and Apparent Potential

The UV–vis and electrochemical experimental data, supported by MD modeling data, clearly show that Eu speciation in chloride salts, and likely that of some other lanthanides, is highly sensitive to the salt’s chlorobasicity, which is governed by the identity and concentration of the salt cations present. A dependence of lanthanide redox thermodynamics on solvent-cation identity has also been reported previously in alkali chloride melts.^15,61,62^ This sensitivity is, for instance, reflected in MD simulations, which show that changes in melt composition strongly reorganize both the inner-sphere chloride coordination of Eu as well as its outer-sphere cation environment. Mg-rich compositions promote chloride bridging and Mg–Cl network formation, which strongly alters the free-energy landscape of Eu solvation and favors configurations compatible with Eu^2+^. The MD results provide a structural interpretation consistent with the electrochemical trend on the chlorine scale. As salt chlorobasicity decreases, evidenced by an independent UV–vis basicity probe showing a composition-dependent absorption shift and a monotonic decrease in optical basicity with increasing MgCl_2_ content relative to LiCl or KCl, the Eu^3+^/Eu^2+^ apparent potential shifts to more positive values. This reflects a stronger oxidizing strength of Eu­(III) and implies that Eu­(III) is less favored (the Eu­(III)-stability boundary is displaced to more oxidizing potentials), such that its stability domain contracts relative to Eu­(II). Together, these trends corroborate a reduction in effective chloride donor strength across the series, and the correlated increase in *E*_app_.

Chemically, these observations are consistent with the picture that Mg^2+^, with its high charge density, polarizes coordinated chloride anions and promotes bridging linkages, thereby lowering the effective availability of Cl^–^ to Eu at a given oxidation state. A similar solvent-cation effect has been observed for other rare-earth ions, which were found to be less strongly solvated by chloride in a CaCl_2_–NaCl melt than in LiCl–KCl because chloride participates in calcium chlorocomplexes.^63^ MD simulations explicitly capture this behavior, showing an increased prevalence of shared chloride ligands between Mg^2+^ and Eu, indicative of chloride participation in extended Mg–Cl connectivity rather than Eu coordinated exclusively through Cl^–^ shared with monovalent cations. This outer-sphere reorganization increases the stability of Eu^2+^ relative to Eu^3+^ and provides a structural interpretation for the positive shift in the Eu^3+^/Eu^2+^ apparent potential observed in Mg-rich (less chlorobasic) melts. In other words, chlorobasicity sets the position of the Eu buffer *E*_app_ on the chlorine scale. Consistent with this interpretation, the Eu^3+^/Eu^2+^ apparent potential increases as salt chlorobasicity decreases. Although these structural descriptors provide insight into the composition-dependent solvation of Eu^3+^ and Eu^2+^, the present simulations do not quantitatively determine their activities or activity coefficients. Establishing a quantitative relationship between molten-salt microstructure, species activity, and redox thermodynamics will require additional free-energy calculations and represents an important direction for future work.

### 4.2 Chlorobasicity Effect on Corrosion

In LiCl-KCl, in situ XAS shows that Eu remains mostly in the Eu^3+^ oxidation state (Figure 8). In contrast, in KCl-MgCl_2_, increasing MgCl_2_ content progressively favors Eu^2+^, with the 60 mol % MgCl_2_ yielding a final XANES spectrum dominated by Eu^2+^ features. Thus, a pronounced reduction of EuCl_3_ is observed by in situ XAS, which cannot be accounted for by bulk thermodynamic predictions or by the measured redox potentials in either LiCl-KCl or KCl-MgCl_2_ (Figure 3), indicating that an additional reactive pathway is operative under the XAS conditions. A likely origin of this pathway is the BN crucible. Rather than acting as an inert container, BN may participate chemically in the melt by serving as a chlorination partner and sacrificial reductant. BN has been observed to react with NiF_2_ to form BF_3_(*g*).^64^ By analogy, the following chloride reaction (eq 6)­

6 $$\mathrm{BN}+3\mathrm{Eu}{\mathrm{Cl}}_{3}\to B{\mathrm{Cl}}_{3}\left(g\right)+3\mathrm{Eu}{\mathrm{Cl}}_{2}+1/2N_{2}\left(g\right)$$

was evaluated using HSC Chemistry (version 6.0), and found thermodynamically favorable at temperatures ofabout 300 °C and higher. The corresponding standard potential for the *E* ^0^ (BCl_3_(*g*), N_2_(*g*)/BN) redox couple is about −0.6 V vs Cl_2_. Thus, the potential scales in Figure 3 indicate that EuCl_3_ can oxidize BN in KCl-MgCl_2_ (60 mol %), leading to formation of EuCl_2_, but cannot do so in LiCl-KCl. For KCl-MgCl_2_ (30 mol %), the melt is slightly more chlorobasic yet likely not enough to drop below −0.6 V/Cl_2_ so BN oxidation by EuCl_3_, and thus EuCl_2_ formation, would still occur, albeit less strongly than at 60 mol % MgCl_2_. While the above reaction is thermodynamically possible, actual evidence of the reaction is missing, and will be the topic of further study.

Consequently, in LiCl-KCl eutectic in contact with BN, Eu remains mostly in the +III oxidation state, because of the relatively high effective chloride donor strength and chloride activity, thermodynamically preventing BN chlorination by EuCl_3_. However, under acidic conditions BN can become chlorinated, acting as a sacrificial reductant. Consequently, increasing the MgCl_2_ content in KCl-MgCl_2_ salts progressively stabilizes Eu^2+^, with the 60% MgCl_2_ mixture yielding predominantly reduced Eu. This result indicates that Mg^2+^, due to its strong Lewis acidity, sequesters chloride ions and effectively lowers minimally polarized Cl^–^ activity. This confirms our earlier hypothesis that chlorobasicity defines a chemical potential for Cl^–^ that governs the redox stability of multivalent metal cations. It also explains why redox buffers like EuCl_3_ (or UCl_3_ in fuel salts) are not expected to behave identically across salt matrices, despite having similar formal oxidation states. That is because their speciation (and their apparent potential) is modulated by the system’s chlorobasicity. Indeed, salt composition and cation identity has been correlated with apparent potentials in molten chloride and oxide melts.^15,18,20,61,65–69^ Bauke and Duffy developed empirical equations for predicting either the basicity or redox ratio for a host of metal cations in alkali silicate and oxide melts.^70^ For nearly all redox pairs reported in their work, lower basicity causes an increase in the M^*n*+^/M^(*n*+*m*)+^ ratio due to the shift in equilibrium relative to atmospheric oxygen and oxide anions in the melt, i.e., the O^2–^/O_2_(*g*) ratio, which describes the oxobasicity for these systems. Empirically, lower a_O2–_ in more acidic salts favors the lower oxidation state of dissolved cations. This reflects the effect that stronger electron donor power from O^2–^ has in more basic melts, where oxyanions act as ligands to stabilize higher charge cations.

A key difference between oxide systems and our chloride salts is that in oxide melt redox studies, there is typically an environment of fixed O_2_ partial pressure, which directly buffers the O^2–^/O_2_(*g*) couple at a defined potential, while the M^*n*+^/M^(*n*+*m*)+^ equilibrium ratio follows changes in that potential, i.e., oxo-basicity. As such, oxo-basicity can mediate redox reaction on its own, with a constant O_2_(*g*) supply to balance electron exchange. Our system has no analogous set Cl_2_(*g*) partial pressure. In the present work, BN is suggested to act as the electron donor, thus reducing agent for Eu^3+^, with efficacy governed by the chlorobasicity dependent lanthanide reduction potential. Chlorobasicity does not act as an independent oxidant/reductant; rather, it sets the apparent potential for the Eu­(III)/Eu­(II) couple and determines whether a given reaction is thermodynamically favorable. This differs from aqueous solution and oxide melt cases, where basicity more directly modulates the stability of solid corrosion products at a fixed external redox potential.

### 4.3 Chlorobasicity as a First-Order Control on Speciation, Redox Buffering, and Corrosion

As shown in Figure 10 and in Figure S5, oxidation kinetics are slower in MgCl_2_-containing salts than in eutectic LiCl-KCl. This behavior is consistent with the stronger Lewis acidity of Mg^2+^, which binds chloride more strongly and lowers its effective activity in the melt. Reduced chloride donating ability is expected to modify the speciation and stability of dissolved chromium chloride species, which may reduce the chromium dissolution rate and contribute to the slower overall corrosion kinetics, although EXAFS data would be needed to further validate this point. In contrast, in LiCl-KCl, chloride electron density is more available for donation to Cr^3+^ (higher *a*_Cl^–^_), stabilizing Cr ions, and the corrosion reaction is faster. After about 100 min of exposure, all salt compositions converge to a similar extent of Eu reduction, indicating that once the interface layer is established near the salt/metal interface, the kinetics become controlled by a common process. This may be the diffusion of reactive species through the transition layer (i.e., Cr diffusion through the Cr-depleted region of the alloy and/or Eu­(III) diffusion through the corrosion-product-enriched interfacial salt region) or depletion of available Eu­(III) oxidant in the near-interface region, regardless of salt composition.

Taken together, our in situ XAS/XRF results support a coherent redox picture in which Eu^3+^ acts as the oxidant for chromium dissolution from Ni-20Cr, with the progressive Eu^3+^ to Eu^2+^ conversion closely tracking the rise of the Cr signal in the melt. Critically, no Cr signal is detected when EuCl_3_ is absent, indicating that Eu^3+^ is required, and thus is the oxidizer, for Cr dissolution under these conditions. In addition, the coupling between the Eu L_3_-edge shift and Cr Kα intensity indicates that chromium oxidation is mediated by the Eu^3+^/ Eu^2+^ redox couple rather than by chloride alone, or impurities. We also note that the presence of BN in the in situ cell can bias Eu speciation in MgCl_2_-rich melts by promoting further Eu^3+^ reduction as chlorobasicity decreases, thereby suppressing the effective oxidizing power of the salt relative to LiCl-KCl under otherwise similar conditions. While BN reduction is certainly present and plays an additional role in the corrosion experiment, in LiCl-KCl, a faster Eu^3+^ to Eu^2+^ conversion is observed even though BN is not expected to reduce EuCl_3_ in this eutectic compared to MgCl_2_-rich melts. This indicates that any BN-induced bias on Eu speciation is minor in our corrosion cells, and the observed redox behavior is governed primarily by the europium and chromium coupling. The underlying point is broadly relevant to MSR environments where salt chlorobasicity can dictate the extent of chlorination/redox activity of other species (such as structural materials, fission products, or getters), thereby shifting buffer speciation and the effective redox window of the salt. As shown schematically and summarized in Figure 12, MD simulations support this interpretation by showing that Mg^2+^-rich melts alter Eu’s outer-sphere solvation through stronger Mg–Cl interactions.

**Figure 12:**
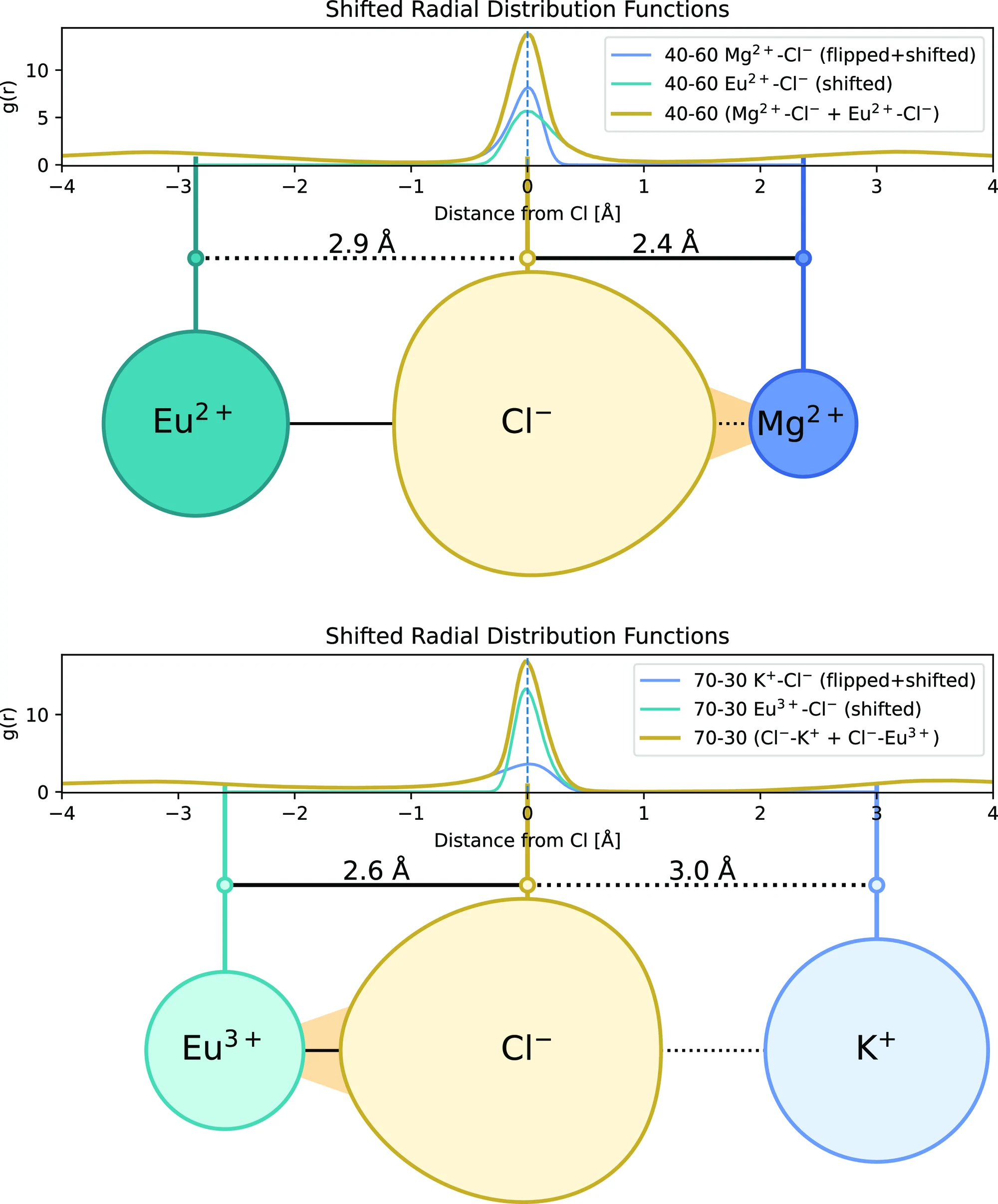
Influence of outer-sphere cations on europium redox speciation in molten chlorides. Selected radial distribution functions shifted by their first peak position to be centered at *r* = 0. For Mg^2+^-Cl^–^ and K^+^-Cl^–^, these were subsequently mirrored around the *y* axis to emphasize the nearest distance around a central Cl- ion. Top: From 1 wt % EuCl_2_ in 40–60 mol % KCl-MgCl_2_, it can be seen that high-charge-density cations (Mg^2+^) strongly polarize and organize chloride into Mg–Cl networks with frequent chloride bridging. This reorganized outer-sphere environment stabilizes coordination motifs compatible with Eu^2+^. Bottom: From 1 wt. EuCl_3_ in 70–30 mol % KCl-MgCl_2_, in contrast, low-charge-density cations (K^+^) interact more weakly with chloride and do not promote extensive network/bridging, leaving chloride coordination more flexible and thereby favoring solvation environments in which Eu^3+^ remains stabilized. This schematic interpretation is supported by MD results, which show that Eu^3+^ preferentially avoids Mg^2+^-rich outer-sphere environments, whereas Eu^2+^ can be accommodated within Mg–Cl networks through shared chloride coordination. See Supporting Information for details on g­(r) calculation including shifted distances.

## Conclusion

This work advances the mechanistic understanding of corrosion in molten chloride salts by directly linking alloy degradation to the Lewis acid–base character of the melt (chlorobasicity) using a first-of-its-kind corrosion cell integrated with in situ X-ray spectroscopy. The coupled XAS/XRF approach enables simultaneous tracking of redox-buffer speciation and alloy dissolution during exposure, providing a direct experimental framework to connect salt chemistry, redox evolution, and corrosion behavior in real time.

Across LiCl-KCl and KCl-MgCl_2_ melts, the results show that salt chlorobasicity governs the speciation of redox-buffer additives such as Eu­(III) and Eu­(II). Moving from relatively chlorobasic LiCl-KCl to more acidic KCl-MgCl_2_ mixtures results in a positive shift of the experimentally measured Eu­(III)/Eu­(II) apparent potential. PIM-MD simulations independently reveal composition-dependent differences in the solvation structures of Eu­(III) and Eu­(II), including changes in Eu coordination and participation in bridged Mg–Cl networks, providing a structural interpretation consistent with this experimentally observed solvent dependence. Together, these results establish chlorobasicity as a predictive chemical descriptor for redox behavior across chloride salt systems.

Under in situ XAS conditions, time-resolved Eu L_3_-edge XANES reveals progressive Eu­(III) reduction in MgCl_2_-containing melts. This behavior is consistent with a chlorobasicity-activated reaction with BN cell components. In less chlorobasic melts, the salt redox chemistry enables BN chlorination, allowing BN to act as a sacrificial reductant/chlorinating agent that promotes Eu­(III) to Eu­(II) conversion, whereas this pathway is not thermodynamically favored in LiCl-KCl. The in situ measurements also demonstrate that Eu­(III) is the primary oxidant driving chromium dissolution from Ni-20Cr under the present conditions. The temporal correspondence between Eu­(III) reduction observed by XAS and Cr accumulation in the melt measured by XRF, together with negligible corrosion in salt without europium, supports Eu­(III)-mediated oxidation of metallic Cr as the dominant corrosion pathway. In this framework, chlorobasicity modulates corrosion kinetics by controlling the oxidizing power of the redox buffer through its effect on Eu speciation.

Beyond its impact on redox-buffer couples such as Eu^3+^/Eu^2+^, salt chlorobasicity should be expected to systematically influence fission-product and fuel speciation in chloride MSRs, because it shifts the relative stability of oxidation states and coordination motifs by changing the effective chloride activity and the melt’s solvation landscape. In practice, this means that the same nominal redox condition (or additive concentration) can correspond to different dominant complexes, activities, and transport properties for key species (such as lanthanides/actinides, noble metals, and transition-metal chlorides), which in turn can strongly modulate corrosion thermodynamics and kinetics, including the driving force for selective dissolution, the stability of soluble corrosion/fission products, and the propensity for interfacial deposition or mass transfer. As a result, chlorobasicity should be treated as a first-order chemical parameter alongside redox potential when interpreting corrosion behavior and when designing mitigation strategies. Critically, the same composition-driven effects are likely to be even more consequential in actinide-containing salts, where multivalent speciation (such as U^3+^/U^4+^, Pu^3+^/Pu^4+^) directly governs fuel chemistry and may couple to structural-material degradation. Therefore, analogous in situ speciation measurements and modeling in actinide-bearing systems are an important next step. In future work, the support of quantitative modeling and experimental mapping of the chlorobasicity-redox potential-speciation relationships would enable predictive design of salt and alloy systems. Additionally, longer-term corrosion studies utilizing these in situ techniques will be essential to fully understand how the corrosion mechanisms evolve from initial dissolution via Eu^3+^ mediated redox reaction and observe the impacts of modified salt chemistry and alloy surface structure.

## Supplementary Material



## Data Availability

The digital data for all figures, tables, charts, and any other media contained in this publication and its associated Supporting Information files will be made accessible on the Zenodo repository, under Digital Object Identifier (DOI): http://dx.doi.org/10.5281/zenodo.22214056.

## Notes

XAS acquisition and fitting procedures; photographs and schematics of the speciation and corrosion cell assemblies (Figures S1, S2); cyclic voltammograms of the Eu^3+^/Eu^2+^ couple (Figure S3); smoothed CVs of the Eu^3+^/Eu^2+^ couple and derivative analysis for redox potential estimation (Figure S4); %Eu­(II) versus Cr XRF signal for Ni-20Cr corrosion in LiCl-KCl and KCl-MgCl_2_ salts (Figure S5); radial distribution functions from MD simulations (Figure S6); molecular dynamics simulation cell compositions (Table S1); born-Mayer-Huggins and charge-dipole potential parameters used in PIM simulations (Tables S2, S3); MATLAB code for Cr XRF peak integration (PDF)
