# Supplementary material for: Redox Buffering and Corrosion in Molten Chloride Salts Dictated by Chlorobasicity
Source: J Am Chem Soc. 2026 Oct 16;148(42):45515–31. doi: 10.1021/jacs.6c11331 (PMC13721176; doi:10.1021/jacs.6c11331)
Supplement: Supplementary file 1 [file ja6c11331_si_001.pdf]

Redox Buffering and Corrosion in Molten Chloride Salts Dictated by Chlorobasicity

Supporting Information

Kailee Buttice[1], Bonita Goh[1], Matthew S. Emerson[2], Ellie Kim[3], Yuxiang Peng[4], Bobby Layne[2], Isabelle Baggenstoss[1], Shea McCarthy[1], George Vukovic[1], Changkyu Kim[1], Jagadeesh Sure[1], Sheng Dai[3,5], Céline Cannes[6], Sylvie Delpech[6], James F. Wishart[2], Santanu Roy[5], Yu-chen Karen Chen-Wiegart[4,7], Adrien Couet*[1]*

[1]Department of Nuclear Engineering and Engineering Physics, University of Wisconsin – Madison, WI 53706, United States

[2]Chemistry Division, Brookhaven National Laboratory, NY 1197, United States

[3]Department of Chemistry, University of Tennessee – Knoxville, TN 37996, United States

[4]Department of Material Science and Chemical Engineering, Stony Brook University, NY 11794, United States

[5]Chemical Sciences Division, Oak Ridge National Laboratory, Oak Ridge, TN 37830, United States

[6]IJCLab, CNRS/IN2P3, Université Paris-Saclay, 91405 Orsay, France

[7]National Synchrotron Light Source-II, Brookhaven National Laboratory, NY 11973, United States

XAS acquisition and fitting procedures: Energy scanning parameters were optimized to prioritize high-resolution near-edge features for Eu L_3-edge XANES, given time constraints resulting from the transient nature of corrosion evolution 10 eV steps from -200 to -30 eV (pre-edge), 2 eV steps from -30 to -10 eV, 0.5 eV steps from -10 to +25 eV (edge region), and 200 eV steps from +25 eV to 10,000 eV (post-edge).

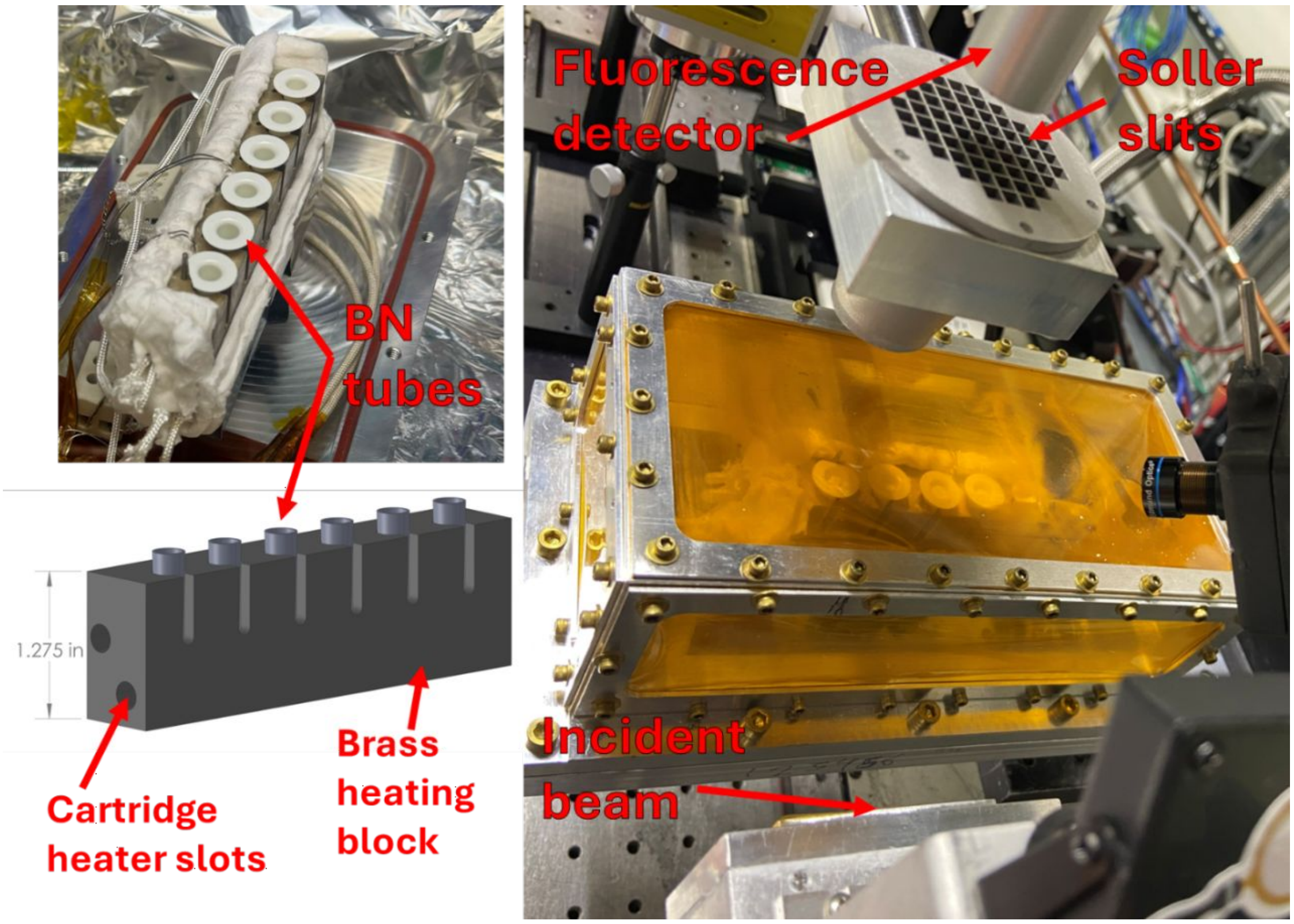

Figure S1: *Setup for measurements of salts contained in BN crucibles. The base of the XAS chamber is shown in the top left and a rendering of the heater block and crucibles is shown for scale in the bottom left. The Kapton-windowed chamber is shown on the beamline in the image on the left. The chamber is positioned such that the crucible being measured is horizontally in line with the incident beam and vertically with the Soller slits and detector.*

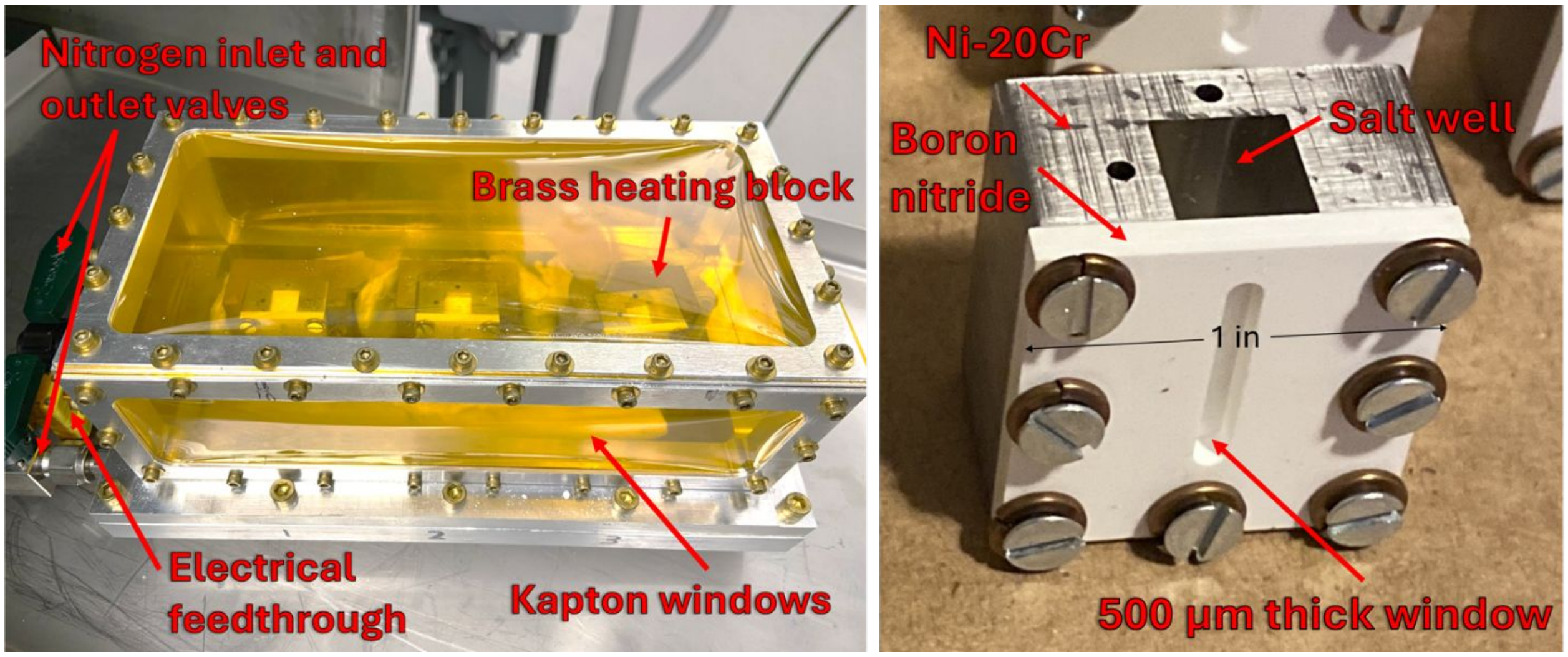

Figure S2: *Chamber (left) and salt containment cell (right) for in-situ corrosion measurements. Each cell loaded into the chamber was heated independently via cartridge heater in its own brass holder*

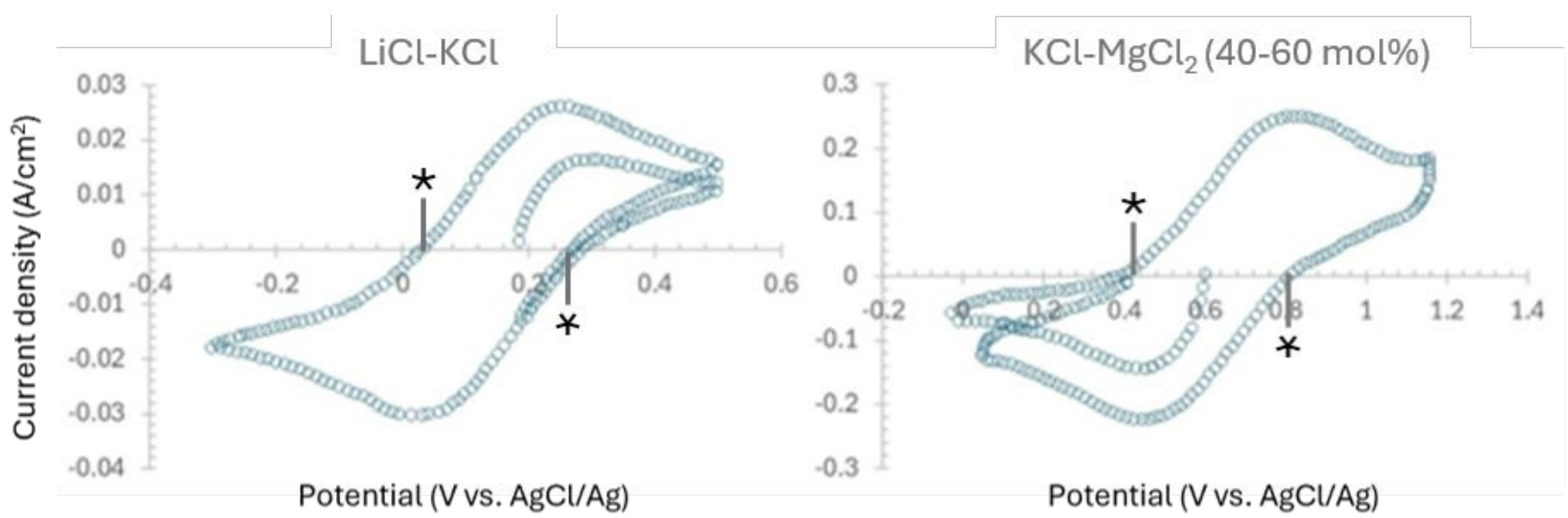

Figure S3: *Cyclic voltammograms of the $Eu^{3+/2+}$ couple in LiCl-KCl and KCl-(60%mol)$MgCl_2$ with a scan rate of 0.1V/s. Potential is plotted vs. AgCl/Ag. The Eu(III)/Eu(II) redox potential can be estimated as the midpoint between the zero-current crossover potentials (marked with asterisks) of the anodic and cathodic sweeps (~0.15 and 0.62 V vs. AgCl/Ag for LiCl-KCl and KCl-60$MgCl_2$ salts, respectively). The difference in salt chlorobasicity results in an estimated 0.47 V difference in Eu(III)/Eu(II) redox potential between the two salts.*

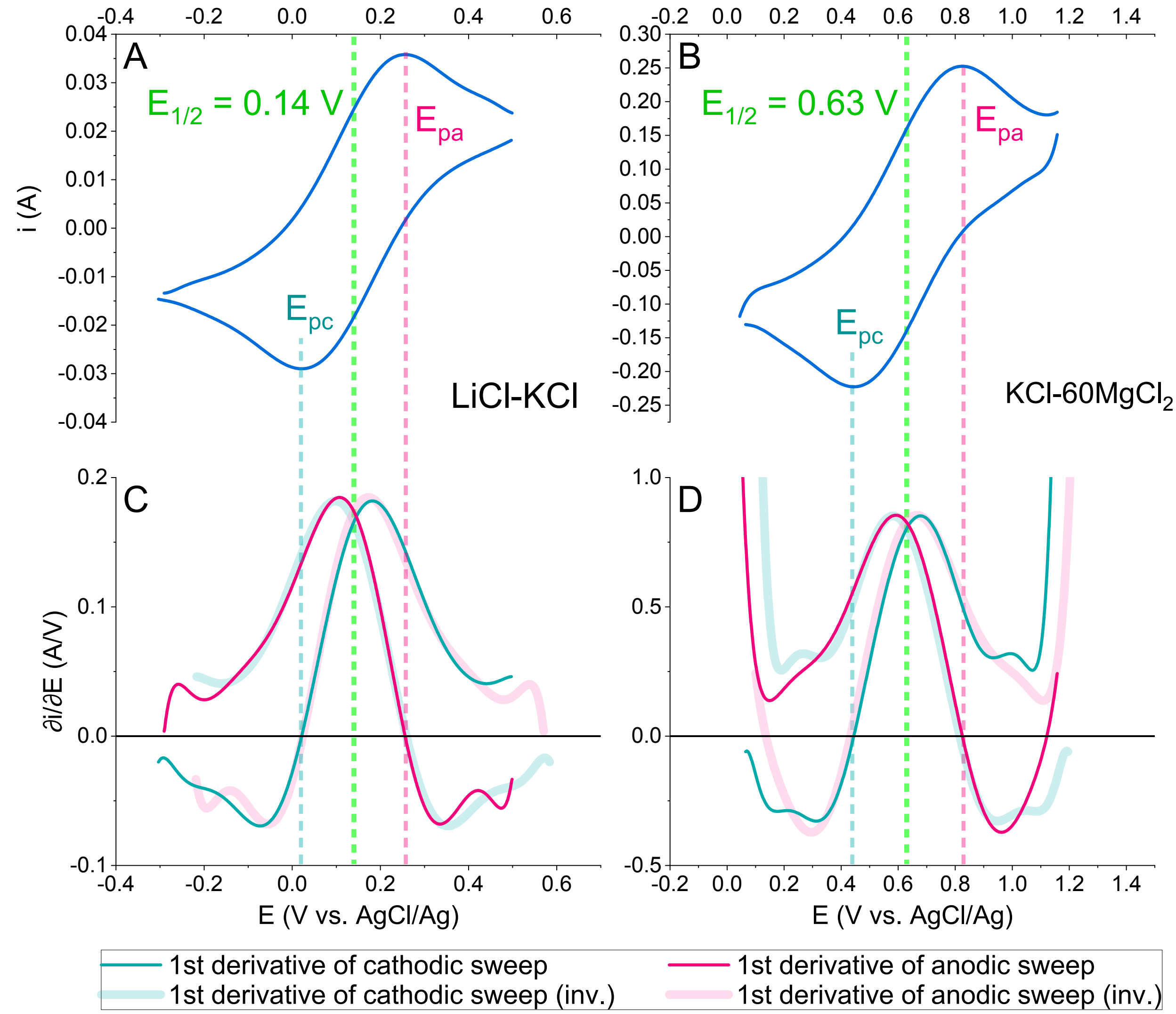

Figure S4: *Smoothed anodic and cathodic sweeps from the cyclic voltammograms in Figure S3 of the* $Eu^{3+/2+}$ *couple in (A) LiCl-KCl and (B) KCl-(60%mol)MgCl*$_2$*. Potential is plotted vs. AgCl/Ag. Derivative plots of the anodic and cathodic branches and their inverses about* $E_{1/2}$ *for (C) LiCl-KCl and (D) KCl-(60%mol)MgCl*$_2$*. The Eu(III)/Eu(II) redox potential can be estimated by calculating* $E_{1/2} = (E_{pa} + E_{pc})/2$ *(~0.14 and 0.63 V vs. AgCl/Ag for LiCl-KCl and KCl-60MgCl*$_2$ *salts, respectively), where* E_{pa} *and* E_{pc} *are the anodic and cathodic peak potentials extracted from the zero-crossing point of the CV first derivatives,* $\partial i/\partial E = 0$*. This estimation method is dependent on the reversibility of the couple, which is validated by the agreement between the CV branch derivatives and their inverses about* $E_{1/2}$*. More details of redox potential estimation by CV may be found elsewhere.[1] The difference in Eu(III)/Eu(II) redox potential between the two salts is an estimated 0.49 V, which is in agreement with the method described in the Figure S3 caption, within 20 mV.*

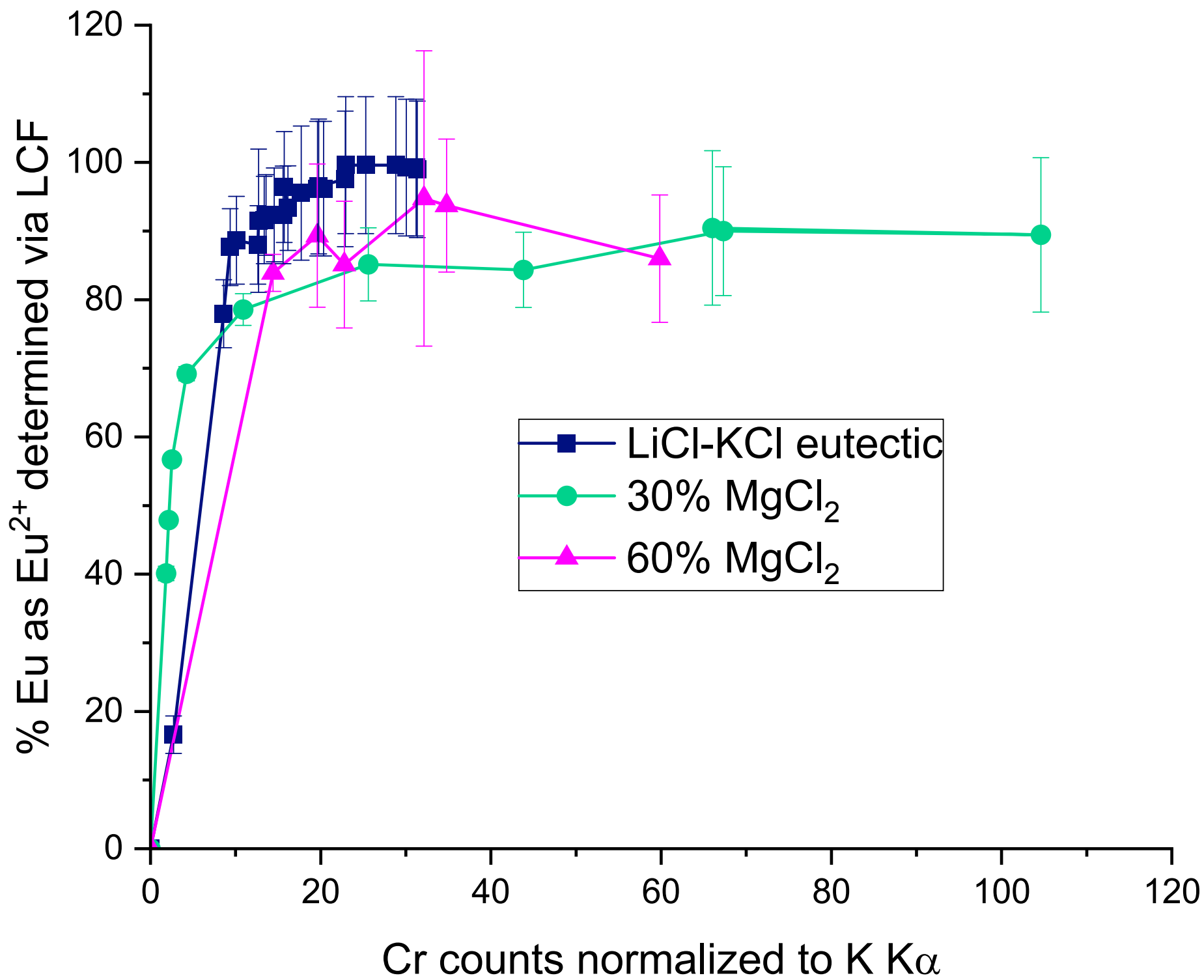

Figure S5: *%Eu as Eu^{2+} during Ni-20Cr alloy exposure to molten LiCl-KCl and KCl-$MgCl_2$ salts containing 1 %wt $EuCl_3$ at 500 °C for the LiCl-KCl salt and 525 °C for the KCl-$MgCl_2$ salts plotted as a function of the amount of Cr in the salt defined by Cr Kα XRF signal normalized to K Kα intensity.*

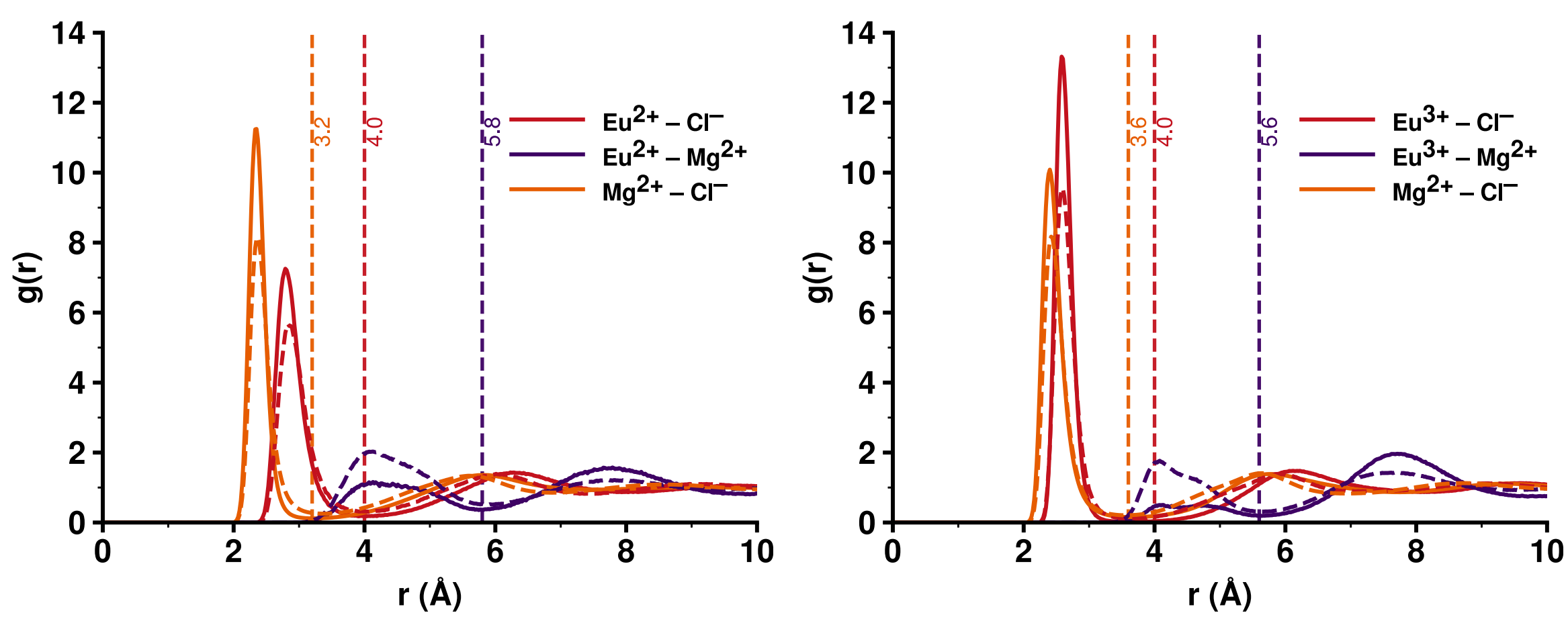

Figure S6: *Selected radial distribution functions, g(r), calculated from MACE MD simulations (left) and PIM MD simulations (right) with either 1 wt% $EuCl_2$ or $EuCl_3$ in both 70-30 mol% (solid lines) and 70-30 mol% (dashed lines) KCl-$MgCl_2$ mixtures at 525 °C, emphasizing cutoffs as vertical lines which were used for free energy analysis in section 3.5. These cutoffs were generated from the first maximum of each corresponding -k_BT ln(g(r)).*

Details for g(r) calculation in Figure 12: Radial Distribution functions were calculated for the Eu^{2+}-Cl^- and Mg^{2+}-Cl^- terms in 1wt% $EuCl_2$ in 40-60 mol% KCl-$MgCl_2$ mixture, and shifted by 2.85 Å and 2.37 Å respectively. Additional g(r)'s for the Eu^{3+}-Cl^- and K^+-Cl^- terms in 1 wt% $EuCl_3$ in 70-30 mol% KCl-$MgCl_2$ mixture were also calculated and shifted by 2.60 Å and 3.00 Å respectively. These g(r)'s were shifted so that the maximum of their first peak was centered at r=0 Angstroms, and the non-Eu-Cl terms were subsequently mirrored around y=0 by performing x=-x of the resulting shifted g(r) in order to emphasize graphically the distances centered around a central "shared" Cl^-. A sum of the two resulting g(r)'s for each system was shown for visualization purposes only.

Table S1: Molecular Dynamics Input Simulation Sizes

System	n_{Eu}	n_{Mg}	n_k	n_{Cl}	n_{total}
1 wt% $EuCl_3$ in 70-30 mol% KCl-$MgCl_2$	12	480	1120	2116	3728
1 wt% $EuCl_2$ in 70-30 mol% KCl-$MgCl_2$	12	480	1120	2104	3716
1 wt% $EuCl_3$ in 40-60 mol% KCl-$MgCl_2$	18	1200	800	3254	5272
1 wt% $EuCl_2$ in 40-60 mol% KCl-$MgCl_2$	18	1200	800	3236	5254

Table S2: *Born-Mayer-Huggins potential parameters used in Polarizable Ion Model simulations performed in this study (all in atomic units). See Metalwalls software information for implementation details.*[2]

Ion Pair	α_{ij}	B_{ij}	C_{ij}^{6}	C_{ij}^{8}	b_{ij}^{6}	b_{ij}^{8}	Ref.
Eu^{3+}-Eu^{3+}	3.0	0.0	32.916	69.006	1.5	1.0	Goloviznina et. al.[3]
Eu^{3+}-Cl^{-}	1.8	238.52	67.884	139.002	1.5	1.0	Goloviznina et. al.[3]
Eu^{3+}-K^{+}	5.0	0.0	19.624	59.787	1.5	1.0	Approximated using Eu^{3+}-Na^{+} from Goloviznina et. al.[3]
Eu^{3+}-Mg^{2+}	5.0	0.0	19.624	59.787	1.5	1.0	Taken as same as Eu^{3+}-K^{+}
Mg^{2+}-Mg^{2+}	5.0	1.0	1.0	10.0	1.7	1.7	Wu et. al.[4,5]
Mg^{2+}-K^{+}	5.0	1.0	0.0	0.0	1.7	1.7	Wu et. al.[4,5]
Mg^{2+}-Cl^{-}	1.732	68.60	10.0	20.0	1.7	1.7	Wu et. al.[4,5]
K^{+}-K^{+}	5.0	1.0	1.0	10.0	1.7	1.7	Wu et. al.[4,5]
K^{+}-Cl^{-}	1.833	208.60	29.4	138.3	1.7	1.7	Wu et. al.[4,5]
Cl^{-}-Cl^{-}	1.797	275.10	140.0	280.0	1.7	1.7	Ishii et. al.,[6] Wu et. al.,[4,5] and Goloviznina et. al.[3]

Table S3. *Charge-Dipole damping parameters used in PIM simulations performed in this study.*

$Charge_i$	$Dipole_j$	b_{ij}^4	$order$	c_{ij}^4	Ref.
Eu^{3+}	Cl^-	1.250	4	1.0	Goloviznina et. al.[3]
Cl^-	Eu^{3+}	1.250	4	-1.0	Goloviznina et. al.[3]
K^+	Cl^-	1.632	4	3.0	Ishii et. al.[6]
Cl^-	K^+	1.632	4	0.917	Ishii et. Al.[6]
Mg^{2+}	Cl^-	1.873	4	2.875	Wu et. al.[4,5]

MATLAB code for Cr XRF peak integration for 1 %wt $EuCl_3$ in LiCl-KCl:

```
clc; clear all; close all;
% K Kα normalization channels and Cr Kα integration channels
K_channels  = 320:340;
Cr_channels = 525:565;
% Load all matching txt files in the current directory
files = dir('Eu_NiCr_LiClKCl_1wtpercEuCl3_insitu_*.txt');
n = numel(files);
% Extract time values from filenames for sorting
times = zeros(n, 1);
for i = 1:n
   nums = regexp(files(i).name, '\d+', 'match');
   times(i) = str2double(nums[1]);
end
% Sort by time
[times, idx] = sort(times);
files = files(idx);
int_counts = zeros(n, 1);
figure; hold on;
for i = 1:n
   data = readmatrix(fullfile(files(i).folder, files(i).name));
   E      = data(:,1);
   counts = data(:,2);
   % Normalize to K Kα peak
   norm_counts = counts ./ max(counts(K_channels));
   plot(E, norm_counts);
   % Integrate Cr Kα region
   int_counts(i) = sum(norm_counts(Cr_channels));
end
hold off;
% Plot Cr dissolution time series
```

```
figure;
plot(times, int_counts, 'o-');
xlabel('Corrosion time (min)');
ylabel('Integrated Cr K\alpha counts (normalized)');
```

References

(1) Espinoza, E. M.; Clark, J. A.; Soliman, J.; Derr, J. B.; Morales, M.; Vullev, V. I. Practical Aspects of Cyclic Voltammetry: How to Estimate Reduction Potentials When Irreversibility Prevails. *J. Electrochem. Soc.* **2019**, *166* (5), H3175. DOI: 10.1149/2.0241905JES.

(2) Marin-Laflèche, A.; Haefele, M.; Scalfi, L.; Coretti, A.; Dufils, T.; Jeanmairet, G.; Reed, S.; Serva, A.; Berthin, R.; Bacon, C.; Bonella, S.; Rotenberg, B.; Madden, P.; Salanne, M. MetalWalls: A Classical Molecular Dynamics Software Dedicated to the Simulation of Electrochemical Systems. *J. Open Source Softw.* **2020**, *5* (53), 2373. DOI: 10.21105/joss.02373.

(3) Goloviznina, K.; Notarangelo, M. C.; Tranchida, J.; Bourasseau, E.; Salanne, M. DFT-Based Polarizable Ion Models for Molten Rare-Earth Chlorides: From Lanthanum to Europium. *J. Phys. Chem. B* **2025**, *129* (3), 1016–1025. DOI: 10.1021/acs.jpcb.4c07931.

(4) Wu, F.; Roy, S.; Ivanov, A. S.; Gill, S. K.; Topsakal, M.; Dooryhee, E.; Abeykoon, M.; Kwon, G.; Gallington, L. C.; Halstenberg, P.; Layne, B.; Ishii, Y.; Mahurin, S. M.; Dai, S.; Bryantsev, V. S.; Margulis, C. J. Elucidating Ionic Correlations beyond Simple Charge Alternation in Molten $MgCl_2$-KCl Mixtures. *Journal of Physical Chemistry Letters* **2019**, *10* (24), 7603–7610. DOI: 10.1021/acs.jpclett.9b02845.

(5) Wu, F.; Sharma, S.; Roy, S.; Halstenberg, P.; Gallington, L. C.; Mahurin, S. M.; Dai, S.; Bryantsev, V. S.; Ivanov, A. S.; Margulis, C. J. Temperature Dependence of Short and Intermediate Range Order in Molten $MgCl_2$ and Its Mixture with KCl. *Journal of Physical Chemistry B* **2020**, *124* (14), 2892–2899. DOI: 10.1021/ACS.JPCB.0C00745.

(6) Ishii, Y.; Kasai, S.; Salanne, M.; Ohtori, N.; Ishii, Y.; Kasai, S.; Salanne, M.; Ohtori, N.; Ishii; Yoshiki; Kasai; Satoshi; Salanne; Mathieu; Ohtori; Norikazu. Transport Coefficients and the Stokes–Einstein Relation in Molten Alkali Halides with Polarisable Ion Model. *MolPh* **2015**, *113* (17–18), 2442–2450. DOI: 10.1080/00268976.2015.1046527.
